# Exploring the Binding Effects of Natural Products and Antihypertensive Drugs on SARS-CoV-2: An In Silico Investigation of Main Protease and Spike Protein

**DOI:** 10.3390/ijms242115894

**Published:** 2023-11-02

**Authors:** Kalliopi Moschovou, Maria Antoniou, Eleni Chontzopoulou, Konstantinos D. Papavasileiou, Georgia Melagraki, Antreas Afantitis, Thomas Mavromoustakos

**Affiliations:** 1 Department of Chemistry, National and Kapodistrian University of Athens, 15771 Athens, Greece; 2 Department of ChemoInformatics, NovaMechanics Ltd., 1046 Nicosia, Cyprus; 3 Department of Chemoinformatics, NovaMechanics MIKE, 18536 Piraeus, Greece; 4 Division of Physical Sciences & Applications, Hellenic Military Academy, 16672 Vari, Greece

**Keywords:** SARS-CoV-2, main protease, spike protein, molecular docking, molecular dynamics simulations, similarity search

## Abstract

In this in silico study, we conducted an in-depth exploration of the potential of natural products and antihypertensive molecules that could serve as inhibitors targeting the key proteins of the SARS-CoV-2 virus: the main protease (Mpro) and the spike (S) protein. By utilizing Induced Fit Docking (IFD), we assessed the binding affinities of the molecules under study to these crucial viral components. To further comprehend the stability and molecular interactions of the “protein-ligand” complexes that derived from docking studies, we performed molecular dynamics (MD) simulations, shedding light on the molecular basis of potential drug candidates for COVID-19 treatment. Moreover, we employed Molecular Mechanics Generalized Born Surface Area (MM-GBSA) calculations on all “protein-ligand” complexes, underscoring the robust binding capabilities of rosmarinic acid, curcumin, and quercetin against Mpro, and salvianolic acid b, rosmarinic acid, and quercetin toward the S protein. Furthermore, in order to expand our search for potent inhibitors, we conducted a structure similarity analysis, using the Enalos Suite, based on the molecules that indicated the most favored results in the in silico studies. The Enalos Suite generated 115 structurally similar compounds to salvianolic acid, rosmarinic acid, and quercetin. These compounds underwent IFD calculations, leading to the identification of two salvianolic acid analogues that exhibited strong binding to all the examined binding sites in both proteins, showcasing their potential as multi-target inhibitors. These findings introduce exciting possibilities for the development of novel therapeutic agents aiming to effectively disrupt the SARS-CoV-2 virus lifecycle.

## 1. Introduction

The COVID-19 pandemic is a global outbreak of the novel coronavirus (SARS-CoV-2). It was first identified in Wuhan, China, in December, 2019, and has since spread to become a global pandemic, affecting all countries and continents. The virus is highly contagious and primarily spreads through respiratory droplets from infected individuals. The virus is a positive-sense single-stranded RNA virus that belongs to the Coronaviridae family [1,2]. The viral genome of SARS-CoV-2 consists of various structural and non-structural proteins, including the spike (S) glycoprotein, membrane protein, envelope protein, nucleocapsid protein, and 16 non-structural proteins (nsp1–16) [3,4,5]. These structural proteins play vital roles in the assembly of new virus particles during infection. Within the viral genome, two open reading frames, ORF1a and ORF1b, encode important protease enzymes: the papain-like protease (PLpro) and main protease (Mpro or 3CLpro). These frames also encode essential components like the RNA-dependent RNA polymerase and endoribonuclease [6,7]. 

**Figure 1:**
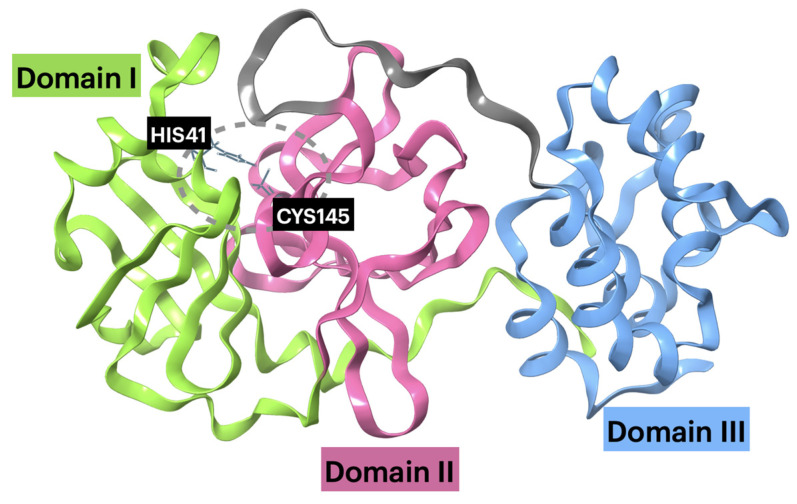
The structure of SARS-CoV-2 Mpro monomer (PDB entry: 6LU7) is depicted in a ribbon representation, where domain I is represented in green, domain II in fuchsia, and the loop region in gray. Additionally, the catalytic dyad, comprising His41 and Cys145, is visually highlighted.

SARS-CoV-2 Mpro operates as a dimer, with each protomer featuring three distinct domains [6,8,9]. Domain I spans residues 8–101, while domain II encompasses residues 102–184, and both exhibit an antiparallel β-barrel structure. Domain III, which covers residues 201–303, consists of five α-helices arranged into a predominantly antiparallel globular cluster. The connection between domain III and domain II is established through an extended loop region spanning residues 185–200. One distinctive characteristic of SARS-CoV-2 Mpro lies in its catalytic dyad, which consists of two key amino acids: Cysteine (Cys145) and Histidine (His41). This dyad plays a pivotal role in the enzyme’s function (see Figure 1). The critical substrate-binding site is nestled within a crevice situated between domain I and domain II. Compounds that bind to this site can effectively inhibit Mpro, preventing it from cleaving its substrate proteins and, thereby, impeding the virus’s replication. Beyond its central role in viral replication, SARS-CoV-2 Mpro was also implicated in modulating the host immune response, adding to its appeal as a target for the development of antiviral drugs.

**Figure 2:**
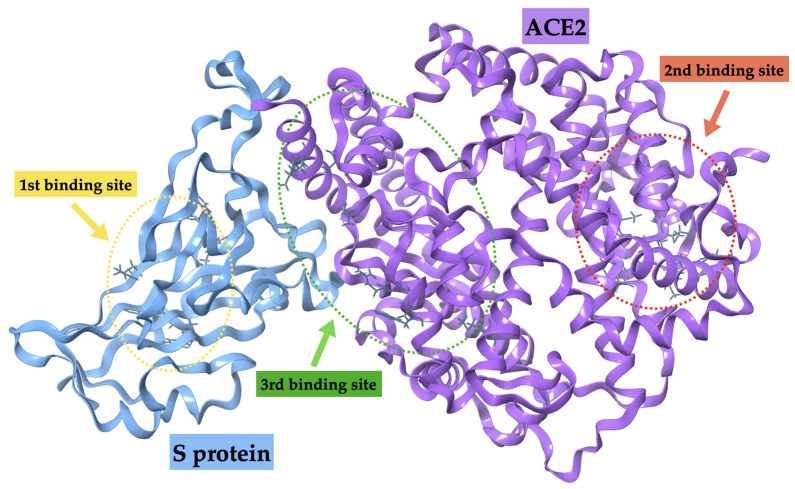
The illustration of the structure of the SARS-CoV-2 S protein bound to ACE2 (PDB entry: 6M0J) through a ribbon representation. In this depiction, the S protein is represented in blue, while ACE2 is highlighted in purple. A yellow circle defines the first binding site, with red representing the second binding site and green representing the third binding site.

The S protein of the SARS-CoV-2 virus is a trimeric structure consisting of three identical subunits that protrude from the surface of the virus. Each subunit is composed of an N-terminal domain, a receptor-binding domain (RBD), and a C-terminal domain. The RBD is the most important part of the spike protein for the virus’s infectivity, as it is responsible for binding to the human ACE2 receptor [10,11]. The interaction between the RBD and the ACE2 receptor is the key event that allows the virus to enter host cells and initiate the replication process. The C-terminal domain of the spike protein is involved in stabilizing the trimeric structure of the spike protein and in modulating the interaction with the host cell. The N-terminal domain was shown to play a role in the formation and stability of the spike protein, as well as in the regulation of its activity [3,4,5]. Overall, the spike protein is a complex structure with multiple functions that are critical for the virus’s infectivity and replication. Structural analysis of the spike protein also provided a basis for the design of neutralizing antibodies that target the RBD and block its interaction with the ACE2 receptor. In Figure 2, we present the structure of the SARS-CoV-2 S protein bound to ACE2 (PDB entry: 6M0J).

Numerous computational studies were dedicated to explore potential drug targets such as Mpro and the S protein against SARS-CoV. Mpro’s pivotal role in viral replication and the S protein’s significance in mediating viral entry make them prime candidates for drug development. Computational investigations employing molecular docking and molecular dynamics (MD) simulations as well as virtual screening were instrumental in unveiling insights into the structural characteristics and dynamic behaviors of these proteins [12,13,14,15,16,17,18,19,20,21,22,23]. These studies aimed to identify novel compounds that can selectively inhibit Mpro’s enzymatic activity and disrupt the binding of the S protein to its host cell receptor.

**Figure 3:**
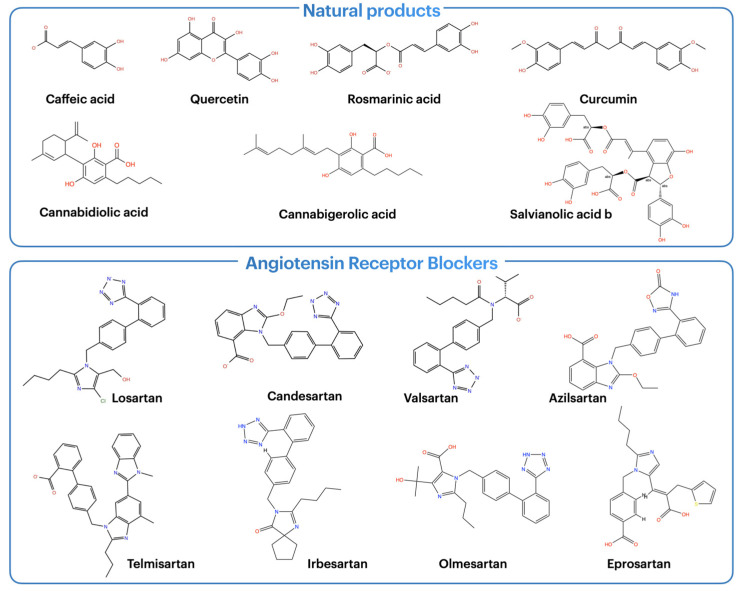
The 2D structures of the studied natural products and antihypertensive drugs.

Natural products have gained significant interest in the field of Rational Drug Design (RDD) due to their various health benefits. They possess anti-inflammatory, antioxidant, and anticancer properties, with recent studies indicating their potential antiviral activity against the SARS-CoV and MERS-CoV viruses [8,9,24,25,26]. Pleiotropic in nature, they have the ability to interact with various cellular targets, making them ideal candidates for combination therapy. Among these compounds, quercetin, curcumin, and caffeic acid are extensively studied for their potential therapeutic use (Figure 3). Quercetin, a flavonoid polyphenolic compound, shows anti-inflammatory and antioxidant effects and is being researched for its potential use as a therapeutic agent [27,28,29,30,31,32]. Curcumin, a polyphenolic compound found in turmeric, has strong anti-inflammatory and antioxidant properties and is being studied for its potential therapeutic use in improving brain function, reducing depression, and fighting inflammation [8,28,29]. Rosmarinic acid, a natural polyphenol found in plants, may have therapeutic potential for conditions like allergies, asthma, arthritis, cancer, and neurological disorders due to its antioxidant, anti-inflammatory, antimicrobial, and neuroprotective properties [33,34]. Caffeic acid, a hydroxycinnamic acid, is studied for its antioxidant, anti-inflammatory, and anticancer properties [35]. Salvianolic acid b, a polyphenolic compound found in Salvia miltiorrhiza, has potential benefits such as antioxidant, anti-inflammatory, and cardioprotective properties. It may also lower blood pressure, improve endothelial function, and possess neuroprotective properties [34,36,37]. Finally, cannabidiolic analogues, similar in structure to cannabidiol (CBD), may have potential therapeutic benefits for conditions such as epilepsy, anxiety, and inflammation [38,39]. The use of natural products in the development of antiviral drugs is an exciting and rapidly growing area of research.

Sartans, also known as angiotensin receptor blockers (ARBs) (Figure 3), are a class of medications commonly used to treat high blood pressure and heart failure. SARS-CoV-2 enters human cells by binding to the angiotensin-converting enzyme 2 (ACE2) receptor, which is expressed on the surface of cells in the lungs, heart, kidneys, and other organs. Some studies suggested that sartans may have the potential to block SARS-CoV-2 from binding to ACE2 and, thus, prevent its entrance in the cell, based on the hypothesis that sartans target the same receptor [40,41,42]. ARBs work by blocking the action of Angiotensin II receptor type 1 (AT1), a hormone that constricts blood vessels and raises blood pressure, by acting as antagonists on the AT1 receptor, which is specific for the Renin–Angiotensin–Aldosterone System (RAAS) and selective for AT1 [43,44,45,46]. 

**Scheme 1:**
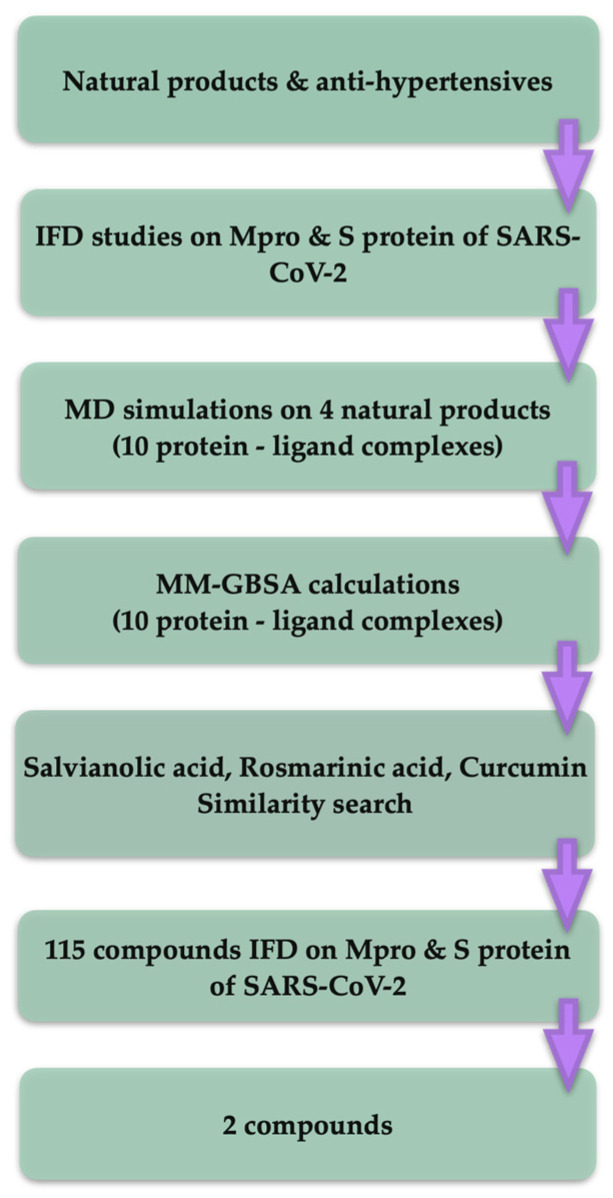
The schematic workflow of the present in silico study.

In this study, we conducted an advanced computational investigation, as outlined in Scheme 1, by combining the techniques of molecular docking and molecular dynamics (MD) simulations. Our goal was to assess the potential of specific natural compounds and antihypertensive molecules to inhibit the interaction between the Mpro enzyme and the S protein bound to human Angiotensin-converting enzyme 2 (ACE2). To pinpoint the most promising compounds, we examined multiple binding sites, including one for the non-structural Mpro and three distinct sites on the structural S protein, as suggested in previous research [21]. We initially employed Induced Fit Docking (IFD) to identify compounds with the strongest binding affinities to the enzymes’ active sites. Subsequently, we subjected these selected “protein-ligand” complexes to detailed MD simulations. Our focus during these simulations was to understand the energetic aspects of molecular interactions, identify potential binding interactions, and evaluate the stability of the “protein-ligand” complexes. To quantify these findings, we performed MM-GBSA calculations. Building on the insights gained from MD results, we further conducted a search for molecules structurally similar to the three most favored ligands—salvianolic acid b, rosmarinic acid, and quercetin. This exploration led us to screen a total of 115 ligands, which we subsequently assessed using IFD at the same binding sites of both proteins. Our efforts culminated in the identification of potential multi-targeting inhibitors based on these comprehensive computational analyses.

## 2. Results

### 2.1. Molecular Docking Results

In this study, our primary objective was to delve into the binding affinities among a carefully chosen selection of natural products and antihypertensive drugs and their respective binding sites on both SARS-CoV-2 MPro and the SARS-CoV-2 S protein.

Within MPro, the binding site consists of specific amino acid residues, primarily found in domain II and the loop region, including His41, Met49, Asn142, Met165, Glu166, Leu167, Pro168, Phe185, and Gln189. Our Induced Fit Docking (IFD) results brought forth three natural products exhibiting the highest binding affinities for SARS-CoV-2 MPro. Notably, rosmarinic acid displayed a binding energy of −9.5 kcal mol^−1^, curcumin presented −8.5 kcal mol^−1^, and quercetin showed −7.8 kcal mol^−1^, underscoring their potential significance for further investigation via MD simulations (Table S1). The docked poses of all structures under study, obtained from IFD on Mpro, are shown in Figure S1.

Furthermore, our IFD analyses revealed intriguing insights into the binding affinities of these compounds at three distinct binding sites on the SARS-CoV-2 Spike protein and ACE2. At the first binding site on the Spike protein (chain E), salvianolic acid b and rosmarinic acid showcased the most favorable binding affinities, registering values of −14.2 kcal mol^−1^ and −11.1 kcal mol^−1^, respectively. The docked poses of all structures under study, obtained from IFD on the S protein’s first binding site, are shown in Figure S2. Shifting our attention to the second binding site targeting the S protein/ACE2 complex after its formation (the binding site is located in ACE2 (chain A)), our findings pointed to salvianolic acid b (−11.1 kcal mol^−1^), quercetin (−10.4 kcal mol^−1^), and rosmarinic acid (−9.9 kcal mol^−1^) as the compounds with the highest binding affinities among those investigated. The docked poses of all structures under study, obtained from IFD on the S protein’s second binding site, are shown in Figure S3. Lastly, we embarked on an exploration of a third binding site, which we refer to as the “new binding site”, located at the intersection of the Spike protein and ACE2. In this context, once again, salvianolic acid b (−10.0 kcal mol^−1^) and rosmarinic acid (−8.3 kcal mol^−1^) displayed favorable binding affinities (Table S2). The docked poses of all structures under study, obtained from IFD on the S protein’s third binding site, are shown in Figure S4.

The outcomes of the Induced Fit Docking (IFD) simulations clearly indicate that sartans do not display the most favorable binding affinity when interacting with both Mpro and the three distinct binding sites of the S protein. These findings strongly imply that within the specific binding sites under investigation, sartans may not be the optimal contenders for establishing robust and enduring molecular complexes. This observation underscores the importance of choosing compounds with superior binding affinity, especially in the context of drug discovery and molecular docking studies.

The natural products that exhibited the most favorable binding affinities were subjected to further investigation through MD simulations, and their docked poses served as the initial conformation for the MD simulations.

### 2.2. Molecular Dynamics Analysis 

#### 2.2.1. Mpro–Ligand Complexes 

**Figure 4:**
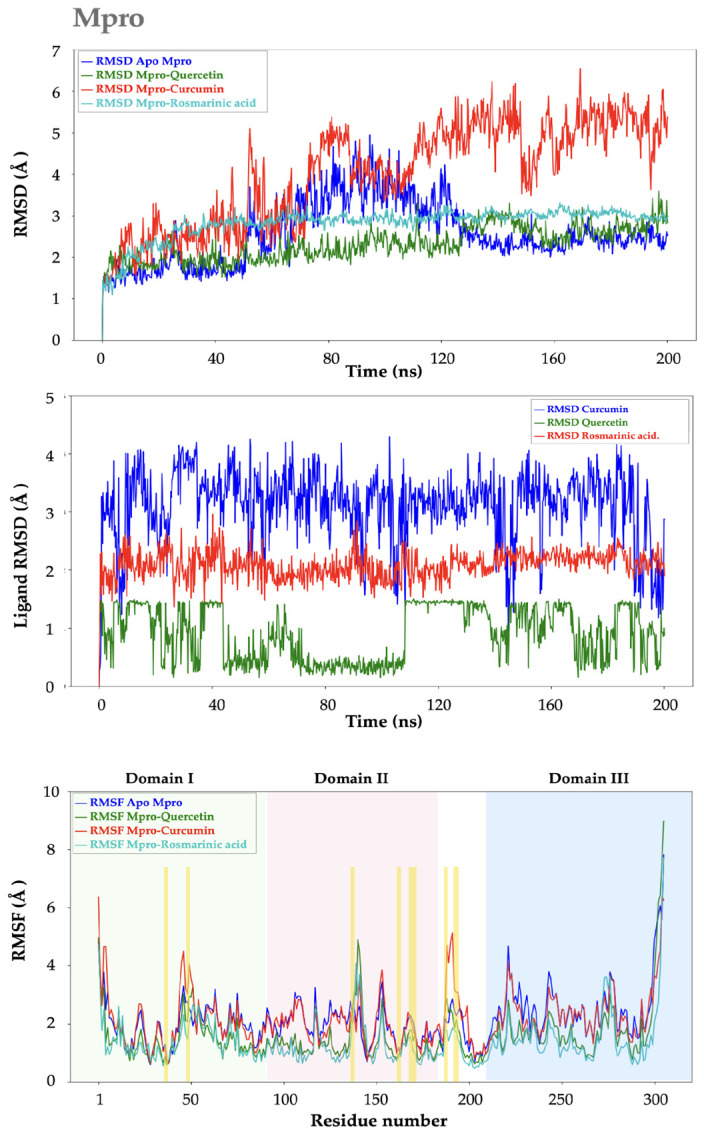
The RMSD of the apo Mpro, the “Mpro—natural products” complexes, and RMSF are aligned to Cα protein atoms. The RMSD of rosmarinic acid, curcumin, and quercetin is aligned with respect to their initial ligand positions. The shaded green, pink, and light blue colors indicate the I, II, and III protein domains, respectively, while yellow lines define the residues in the binding site.

The RMSD of the Mpro–rosmarinic acid complex reveals stable conformational behavior throughout the entire simulation time, with an average RMSD value of 2.8 ± 0.4 Å for the complex and 2.1 ± 0.2 Å for rosmarinic acid (Figure 4). In contrast, the apo form of Mpro exhibits fluctuations between 50 and 120 ns, with an average value of 2.5 ± 0.8 Å. Additionally, the RMSF demonstrates a more stable system in comparison to the apo form of Mpro (Figure 4). 

**Figure 5:**
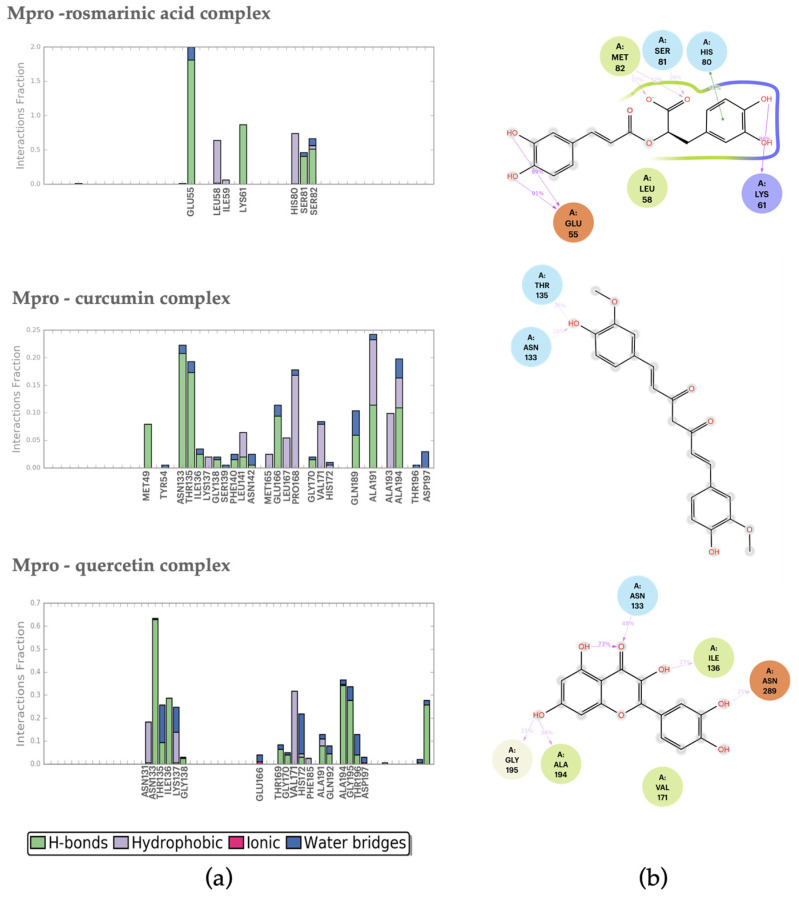
(**a**) The interactions analysis for the Mpro–ligand complexes over the last 40 ns of simulation. The Mpro–ligand interactions are categorized into four types: hydrogen bonds (green color), hydrophobic interactions (purple color), ionic interactions (magenta color), and water bridges (blue color). (**b**) A schematic of detailed ligand atom interactions with the Mpro residues. Only interactions that occurred more than 15.0% of the analyzed simulation time are illustrated.

To obtain a more profound understanding of the stability in the ligand–protein complexation, we closely examined the interactions between Mpro and rosmarinic acid during the final 40 ns of the simulation, a period where the system had reached equilibrium. Our analysis revealed the formation of numerous hydrogen bonds, with the most predominant interactions involving Glu55, Lys61, Ser81, and Met82 residues (H bonds) as well as Leu58 (hydrophobic interactions). Additionally, a pi–pi stacking interaction was observed with His80. Furthermore, water bridges formed with residues Ser81 and Met82, underscoring the crucial stabilizing role of these interactions within the complex (Figure 5), as captured in the Mpro–rosmarinic acid centroid structure deduced by clustering analysis (Figure S5). The conformation of this complex significantly differs in comparison to the initial docked pose (Figure S1), where rosmarinic acid primarily interacts with residues Thr25, His41, Cys44, and Ser46, indicative of this molecule’s significant flexibility and movement within the binding site. However, it is noteworthy that the initial binding pose closely resembles the one proposed from docking calculations by Shahhamzehei et al. [22]. This is also indicated by comparing both conformations (Figure S6). For a comprehensive evaluation of the “protein-ligand” complex, we employed molecular mechanics/generalized Born surface area (MM-GBSA) calculations to estimate the binding affinity and energetics of the interaction. Our MM-GBSA analysis yielded a highly favorable binding free energy of −45.2 ± 3.6 kcal mol^−1^, indicating a robust and stable interaction between rosmarinic acid and Mpro (Table 1). Our findings align with those of Shahhamzehei et al., where they established that rosmarinic acid displayed a binding affinity (Kd) of 15.47 μM to SARS-CoV-2 Mpro using microscale thermophoresis analysis. Additionally, their research demonstrated that rosmarinic acid binds to SARS-CoV-2 Mpro with an IC50 value of 9.43 μM [22].

The trajectory analysis of the Mpro–curcumin complex reveals conformational flexibility throughout the entire simulation time, with a high average RMSD value of 4.0 ± 1.2 Å for the protein and 3.1 ± 0.6 Å for curcumin (Figure 4). This behavior is consistent with the RMSF analysis, which shows notable fluctuations (Figure 4). This observation is further supported by the interaction analysis with Mpro, as curcumin predominantly interacts with the residues Asn133 and Thr135 (Figure 5), also highlighted in the complex centroid via clustering analysis (Figure S5). This centroid structure underscores curcumin’s flexibility inside the binding site, compared to the initial complex’s conformation, since the interactions with residues Thr25, His41, and Asn142 are no longer observed (Figure S1). This is also indicated by superimposing both conformations (Figure S6). Interestingly, despite the observed instability, the MM-GBSA calculations indicated a total free energy value of −47.5 ± 4.0 kcal mol^−1^, indicating a strong and consistent interaction between Mpro and curcumin (Table 1). This observation is in agreement with the in vitro and in silico study of Bahum et al., where curcumin was identified as one of the top five most potent inhibitors of SARS-CoV-2 Mpro. Notably, this finding holds even though curcumin exhibited some degree of mobility during the simulation period [23].

The MD analysis of the Mpro–quercetin complex unveiled a persistent interaction between quercetin and Mpro. The RMSD of the Mpro–quercetin complex indicated a stable system throughout the entire simulation time, except for 125–160 ns, where some fluctuations were observed. The average RMSD values were 2.3 ± 0.4 Å for the complex and 0.9 ± 0.5 Å for quercetin. It is worth noting that the RMSD analysis for the ligand exhibited small fluctuations, possibly due to the ligand’s compact structure. The RMSF also demonstrated overall stability, with the exception of a fluctuation in residues 140–145 in domain II, even though these residues are not part of the binding site, compared to the apo form of the S protein. Furthermore, our analysis of the interactions between Mpro and quercetin revealed the formation of multiple hydrogen bonds, hydrophobic interactions, and water bridges, with notable dominance observed with residues Asn133, Ile136, Ala194, Glu195, and Asp289 (Figure 5), also illustrated in the predominant conformation of the Mpro–quercetin complex obtained through clustering analysis (Figure S5). Additionally, the MM-GBSA calculations underscored the strength of the binding, as evidenced by the low total energy value of −33.8 ± 5.2 kcal mol^−1^ (Table 1). This aligns with the findings of Bahun et al., which provided compelling evidence of quercetin’s potency as an inhibitor of SARS-CoV-2. This research confirmed this efficacy, revealing an IC50 of 23.4 μM [23].

All three compounds—rosmarinic acid, curcumin, and quercetin—demonstrated favorable interactions with Mpro, as indicated by their MM-GBSA binding free energy values. Rosmarinic acid exhibited structural stability and established multiple interactions, including water bridges. Quercetin’s complex with Mpro is characterized by a profile indicating reduced flexibility and the formation of several crucial interactions with a plethora of residues. In contrast, curcumin exhibited conformational instability throughout the simulation, preventing the establishment of a strong interaction with the Mpro binding site. In conclusion, our discoveries strongly underscore the promise of rosmarinic acid and quercetin as potential inhibitors against SARS-CoV-2 Mpro. This is substantiated by the robust interactions they form and the highly favorable binding energy values they exhibit. Moreover, these findings seamlessly align with earlier in vitro studies, further affirming the potent inhibitory properties of these natural compounds [22,23]. 

#### 2.2.2. S protein–Ligand Complexes 

**Figure 6:**
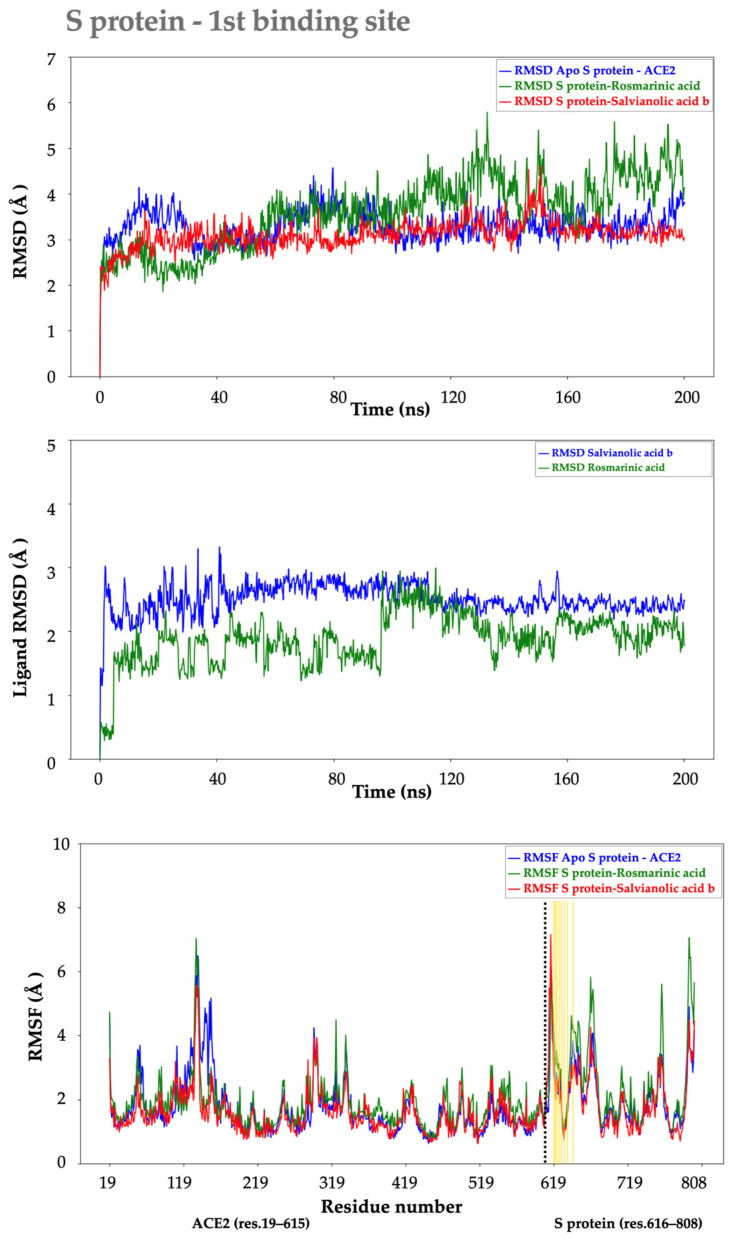
The RMSD and RMSF of Cα protein of apo S protein bound to ACE2 and each docked complex of S protein and salvianolic acid b, as well as rosmarinic acid, define the residues in the binding site with yellow lines. The RMSD of salvianolic acid b and rosmarinic acid is aligned with respect to their initial ligand positions.

**Figure 7:**
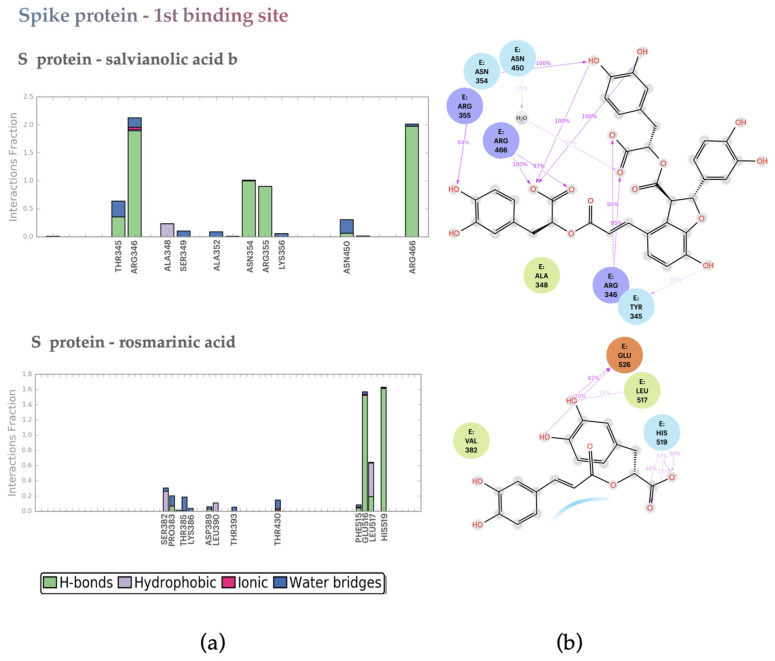
(**a**) The interactions analysis for the S protein–ligand complexes for the first binding site over the last 40 ns of simulation. The S protein–ligand interactions are categorized into four types: hydrogen bonds (green color), hydrophobic interactions (purple color), ionic interactions (magenta color), and water bridges (blue color). (**b**) A schematic of detailed ligand atom interactions with the S protein residues. Only interactions that occurred more than 15.0% of the analyzed simulation time are illustrated.

In our investigation of the initial binding site interaction between the SARS-CoV-2 S protein and salvianolic acid b, we conducted a 200 ns MD simulation to gain deeper insights into their dynamic interplay. The RMSD of the S-protein–salvianolic acid b complex indicates the formation of a stable complex throughout the entire simulation time, with the only exception being 150–160 ns, where some fluctuations are observed (Figure 6). When compared to the apo form of the S protein, which exhibited an average RMSD value of 3.2 ± 0.4 Å, in its complex under study with salvianolic acid b, the protein displayed an average RMSD value of 3.1 ± 0.3 Å, indicative of a system that is conformationally stable. The RMSD of the salvianolic acid b exhibited convergence with an average RMSD value of 3.7 ± 0.4 Å and some minor fluctuations from 0 to 40 ns. In addition, the RMSF analysis for the S protein–salvianolic acid b complex demonstrated slight fluctuations near residue 619, but it generally exhibited behavior similar to that of the apo form (Figure 6). Furthermore, our analysis unveiled the formation of several crucial interactions, which include multiple hydrogen bonds, hydrophobic interactions, and water bridges with various residues, with notable dominance observed with Thr345, Arg346, Asn354, Arg355, Asn450, and Arg466 (Figure 7). These interactions collectively underscore the robust and stable nature of the S protein–salvianolic acid b complex, displayed in the cluster centroid conformation of the S protein–rosmarinic acid complex (Figure S7), and are preserved with respect to the initial complex’s conformation (Figure S2). However, differences in conformations are present when comparing both superimposed structures (Figure S8). Additionally, our MM-GBSA calculations yielded a total free energy value for the system of −56.5 ± 4.9 kcal mol^−1^, further affirming the strength and stability of this interaction (Table 1). Overall, our findings indicated that the binding between salvianolic acid b and the SARS-CoV-2 S protein at the first binding site leads to the formation of a stable complex, despite some fluctuations observed in the MD analysis. Finally, in the study conducted by Hu et al., salvianolic acid b was found to exhibit the highest binding affinity among the compounds tested, displaying a remarkable affinity for the 2019-nCoV spike pseudoviruS. Moreover, salvianolic acid b demonstrated the most potent anti-2019-nCoV pseudovirus effect, effectively inhibiting the entry of the pseudovirus into ACE2h cells [37].

Continuing our exploration of the S protein’s interaction with rosmarinic acid through MD simulations, we uncovered intriguing dynamics. The RMSD of the S protein–rosmarinic acid complex revealed a consistently more flexible system throughout the entire simulation. Specifically, numerous fluctuations were observed, leading to an average RMSD value of 3.6 ± 0.8 Å, higher than the apo form’s value of 3.2 ± 0.4 Å (Figure 6). In contrast, rosmarinic acid itself exhibited an average RMSD value of 1.9 ± 0.7 Å, albeit with noticeable fluctuations. The RMSF analysis showed several pronounced peaks, further emphasizing the heightened dynamics in comparison to the apo form, which is consistent with the RMSD results (Figure 6). An analysis of the interactions during this stable period revealed dominant interactions with specific residues, primarily Glu516, Leu517, and His519 (Figure 7). These interactions suggested that rosmarinic acid slightly shifted from its initial position, as evidenced in the centroid conformation of the S protein–rosmarinic acid complex (Figure S7). This was also evidenced in comparison to the initial conformation, where interactions with residues Val341, Arg346, Ala348, Ser349, Ser399, and Asn450 were observed (Figure S2). This was also indicated by comparing both superimposed conformations (Figure S8). Finally, our calculation of the total free energy of the system via MM-GBSA yielded a value of −41.2 ± 4.4 kcal mol^−1^. This value reflected the overall energetics of the system and suggested a favorable interaction between rosmarinic acid and the S protein.

**Figure 8:**
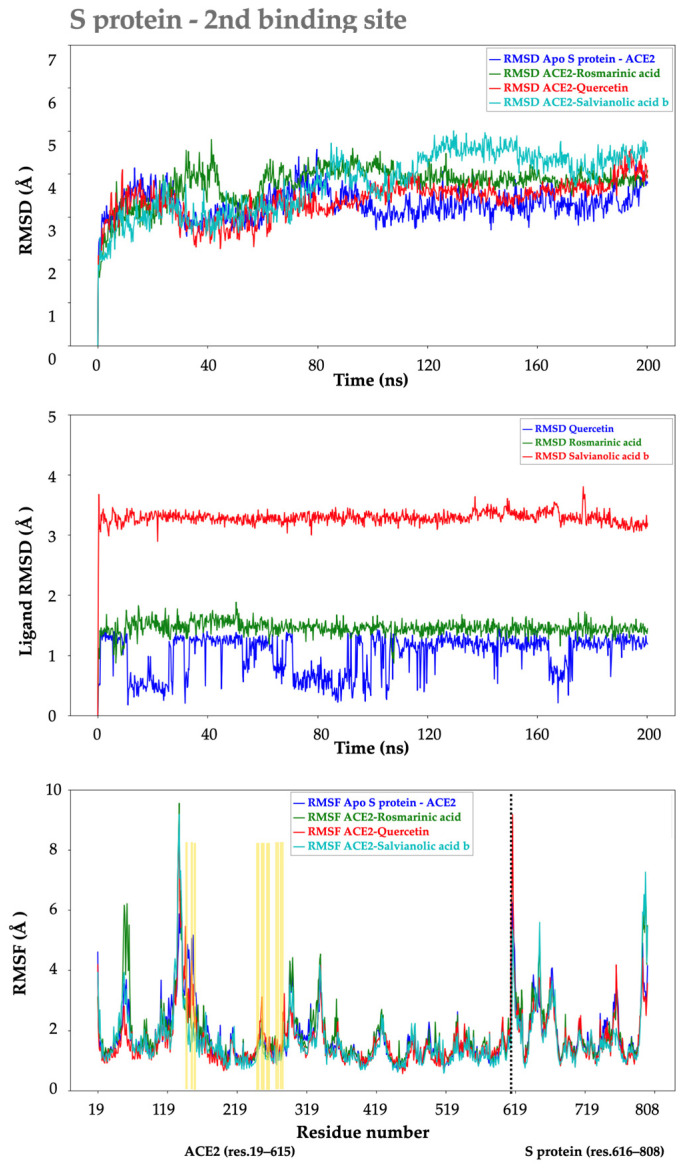
The RMSD and RMSF of Cα protein of apo S protein bound to ACE2 and of each docked complex of S protein bound to ACE2 and salvianolic acid b, quercetin, and rosmarinic acid, define the residues in the binding site with yellow dashes. The RMSD of salvianolic acid b, quercetin, and rosmarinic acid is aligned with respect to their initial ligand positions.

**Figure 9:**
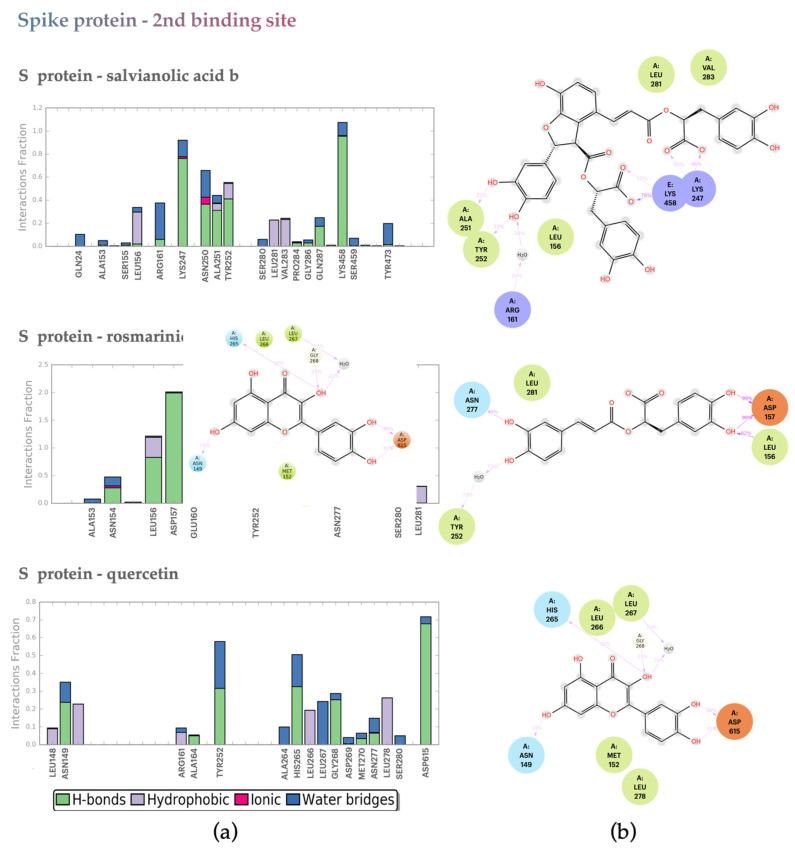
(**a**) The interactions analysis for the S protein–ligand complexes for the second binding site over the last 40 ns of simulation. The S protein–ligand interactions are categorized into four types: hydrogen bonds (green color), hydrophobic interactions (purple color), ionic interactions (magenta color), and water bridges (blue color). (**b**) A schematic of detailed ligand atom interactions with the S protein residues. Only interactions that occurred more than 15.0% of the analyzed simulation time are illustrated.

In our investigation of the second binding site, we thoroughly analyzed the SARS-CoV-2 S protein’s interaction with salvianolic acid b. The RMSD analysis of the S protein–salvianolic acid b complex revealed a flexible system throughout the entire simulation, characterized by a higher average RMSD value of 3.7 ± 0.7 Å when compared to the RMSD value of the apo form, which averaged 3.2 ± 0.4 Å (Figure 8). In contrast, the RMSD (3.2 ± 0.4 Å) analysis of salvianolic acid b at the outset of the simulation indicated conformational changes early in the simulation, which subsequently stabilized over time. Moreover, the RMSF analysis of the complex under investigation demonstrated significant fluctuations in certain residues, underscoring the inherent flexibility of these specific amino acid positions (Figure 9). It is noteworthy that these fluctuating residues were not directly involved in the binding site of the complex nor did they form any interactions. Furthermore, our analysis of the complex’s interactions depicted in the cluster centroid revealed the formation of several interactions with residues Arg161, Lys247, Ala251, and Tyr252, situated near ACE2 (chain A), as well as Lys458, located within the S protein (chain E) (Figure 9 and Figure S9). These interactions differed from the initial complex’s conformation, indicating the slight shift in placement within the specific site, as interactions with residues Glu150, Asn154, Leu156, Ala251, Tyr252, Asn277, and Ser 280 (Figure S3) were no longer observed. This was also indicated by comparing their superimposed conformations (Figure S10). Finally, the MM-GBSA calculations affirmed the stability of the complex, as evidenced by a total free energy value of −42.6 ± 5.8 kcal mol^−1^ (Table 1).

The analysis of the RMSD for the S protein–quercetin complex revealed several noticeable fluctuations, particularly evident between 80 and 200 ns. The average RMSD value for the complex was equal to 3.4 ± 0.4 Å, which closely mirrors the RMSD value for the apo form of the S protein, averaging 3.2 ± 0.4 Å (Figure 8). In contrast, the quercetin molecule itself exhibited an average RMSD value of 1.0 ± 0.3 Å, displaying fluctuations throughout the entire simulation duration. Furthermore, the RMSF analysis highlighted the flexibility of certain residues, despite that these residues were neither part of the binding site nor formed any predominant interactions with the specific ligand. Several types of interactions, including hydrogen bonds, hydrophobic interactions, ionic interactions, and water bridges, were observed. The most prominent interactions occurred with residues Asn149, His265, Gly268, and Asp615 (Figure 9), as illustrated by the longest living cluster of the identified S protein–quercetin complex (Figure S9). The total free energy of the system was quantified at −40.9 ± 6.1 kcal mol^−1^ (Table 1). 

Finally, we conducted MD simulations to investigate the S protein–rosmarinic acid complex at the second binding site. The RMSD analysis of the S protein–rosmarinic acid complex revealed noteworthy fluctuations, primarily observed until 80 ns. However, from 80 to 200 ns, the system maintained stability, with an average RMSD value of 3.7 ± 0.4 Å (Figure 8). This suggests that the complex reached a stable conformation during this later period. In contrast, rosmarinic acid itself appeared very firmly placed at the binding site, with an average RMSD value of 1.4 ± 0.1 Å throughout the simulation, indicating its consistent conformational stability within the protein. Additionally, the RMSF analysis of the studied complex closely resembled that of the apo S protein, with the exception of some noticeable fluctuations observed in residues 38–42. These fluctuations in this specific region were the only discernible deviations from the overall pattern of stability observed in the complex. The interaction analysis exposed several formed interactions, encompassing hydrogen bonds, hydrophobic interactions, ionic interactions, and water bridges. Among these interactions, those with residues Asn157, Leu156, and Asn277 were the most predominant (Figure 9), also captured by clustering analysis of the S protein–rosmarinic acid complex (Figure S9). The overall stability of the system was affirmed by the total free energy value of −41.0 ± 3.8 kcal mol^−1^. 

**Figure 10:**
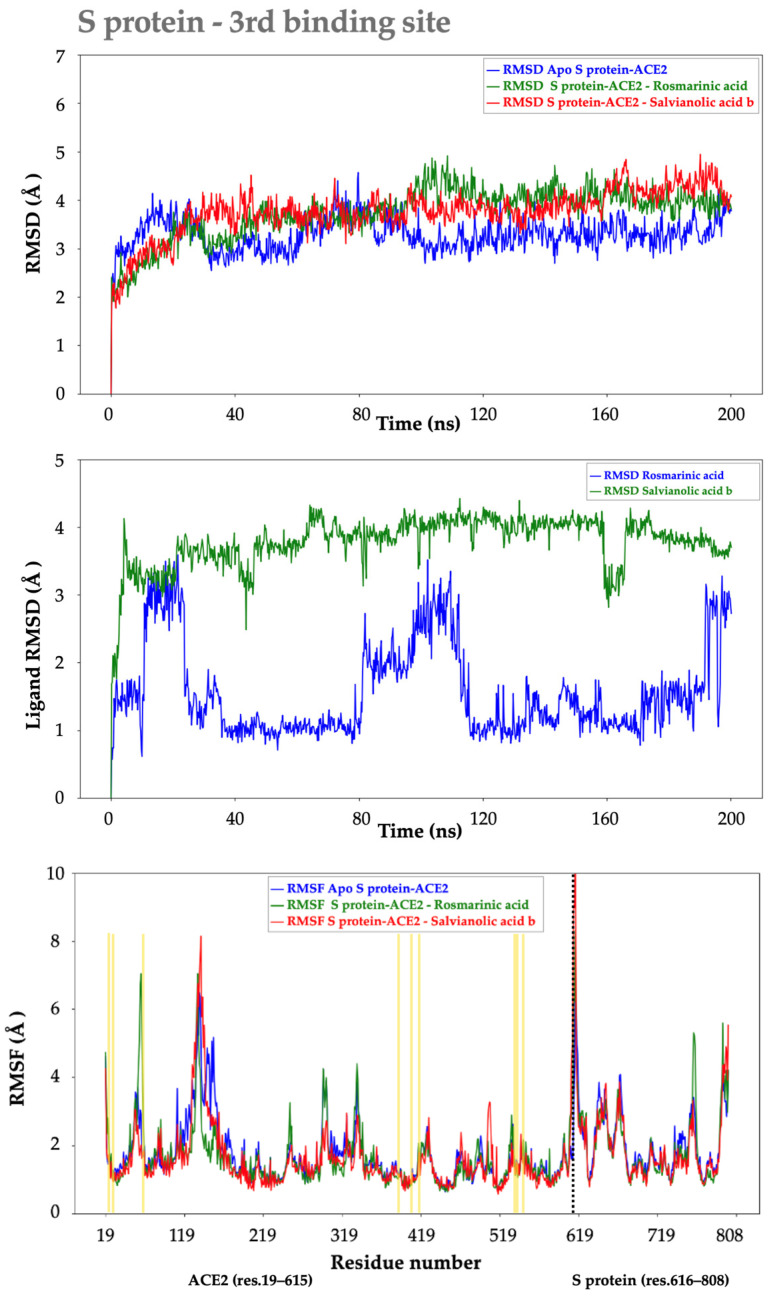
The RMSD and RMSF of Cα protein of the apo S protein and each docked complex of S protein bound to ACE2, salvianolic acid b, and rosmarinic acid (second binding site) define the residues in the binding site with yellow dashes. The RMSD of salvianolic acid b and rosmarinic acid is aligned to ligand.

**Figure 11:**
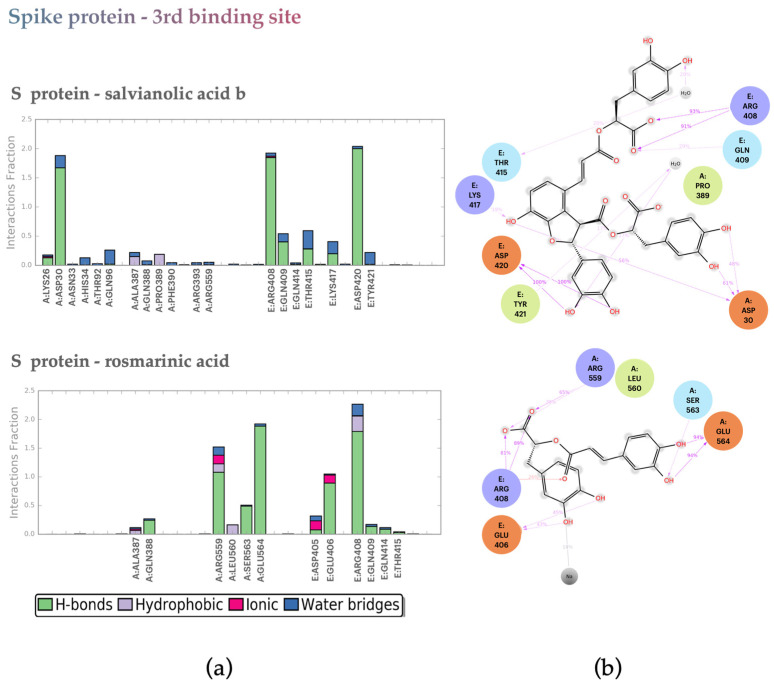
(**a**) The interactions analysis for the S protein–ligand complexes for the third binding site over the last 40 ns of simulation. The S protein–ligand interactions are categorized into four types: hydrogen bonds (green color), hydrophobic interactions (purple color), ionic interactions (magenta color), and water bridges (blue color). (**b**) A schematic of detailed ligand atom interactions with the S protein residues. Only interactions that occurred more than 15.0% of the analyzed simulation time are illustrated.

The interactions between the S protein and salvianolic acid b, as well as the S protein and rosmarinic acid, at the third –and final– binding site of the S protein were also examined. The RMSD analysis of the S protein–salvianolic acid b complex showcased protein stability, characterized by an average RMSD value of 3.8 ± 0.5 Å throughout the simulation (Figure 10). Salvianolic acid b itself exhibited an average RMSD of 3.7 ± 0.4 Å, with noteworthy fluctuations occurring at specific time points, namely, 20, 70, 80, and 100 ns and during the interval from 155 to 165 ns. Furthermore, the RMSF analysis of this complex closely mirrored the behavior of the apo S protein, with the exception of the more pronounced fluctuations observed in residues 115 and 600 (Figure 10). These particular residues displayed bolder variations compared to the overall pattern of stability observed in the complex. Our interactions analysis unveiled several types of interactions, including hydrogen bonds, hydrophobic interactions, ionic interactions, and water bridges, involving the residues constituting the binding site. Particularly noteworthy were the predominant interactions formed with residues Arg408, Gln409, Thr415, Lys417, Asp420, and Tyr421 in the S protein (chain E), as well as residue Asp30 in ACE2 (chain A), contributing to a robust interaction pattern (Figure 11), as captured in the centroid S protein–salvianolic acid b complex conformation (Figure S11). However, a slight shift relative to the initial conformation was observed, since the interactions with residues Ala386, Ala387, Arg408, and Arg559 were not maintained (Figure S4). This was also highlighted by comparison of both conformations (Figure S12). The total free binding energy of the system, determined through MM-GBSA calculations, was calculated as equal to −35.3 ± 5.1 kcal mol^−1^, underscoring the favorability of complex formation (Table 1).

The RMSD analysis of the S protein–rosmarinic acid complex indicated conformational protein flexibility, characterized by an average RMSD value of 3.7 ± 0.5 Å throughout the simulation (Figure 10). In contrast, the RMSD values for rosmarinic acid itself exhibited an average of 1.6 ± 0.7 Å, with most changes observed during specific time intervals, namely, from 80 to 110 ns and from 190 to 200 ns. Moreover, the RMSF analysis of this complex closely mirrored the behavior of the apo S protein, with a few conspicuous exceptions. The most pronounced fluctuations involved residues 45 and 600, representing deviations from the overall pattern of stability observed in the complex. To elucidate ligand selectivity and interaction patterns, we identified the total number of “protein-ligand” contacts from the overall interaction profile during the last 40 ns of the simulation. Multiple interactions, including hydrogen bonds, hydrophobic interactions, ionic interactions, and water bridges, involving the residues within the binding site were identified. The most prominent interactions of rosmarinic acid were observed with S protein residues Glu406 and Arg408, as well as Arg559, Ser563, and Glu564 in ACE2 (Figure 11 and Figure S11), no longer involving Gln96 with respect to the initial conformation (Figure S3), which indicates rosmarinic acid’s conformational change during the simulation, as indicated by their superimposed structures (Figure S12). Finally, the total free energy value was calculated as equal to −40.4 ± 9.1 kcal mol^−1^ (Table 1).

Based on the comprehensive MD analysis conducted for the three binding sites, we can confidently conclude that the three natural products, salvianolic acid b, rosmarinic acid, and quercetin, exhibited strong affinity toward the SARS-CoV-2 Spike protein, forming complexes characterized by stability, robustness, and favorable binding energetics. These findings are significant and suggest the potential utility of these natural products in the development of anti-COVID 19 that targeting the SARS-CoV-2 Spike protein. Further research and exploration of these natural compounds may provide valuable insights into their role in combating viral infections.

### 2.3. Similarity Test Results

Based on the aforementioned findings, the similarity search conducted using the Enalos Suite resulted in the identification of 115 compounds with structures similar to salvianolic acid b, rosmarinic acid, and quercetin. These compounds were then subjected to molecular docking calculations against the Mpro enzyme and the S protein at the aforementioned binding sites.

For the Mpro enzyme, the docking results showed that compounds with CIDs 131676018, 502233, 7439584, 125990, and 24812412 exhibited strong binding affinities, ranging from −16.5 to −15.1 kcal mol^−1^ (Table S3). For the first binding site of the S protein that is located on the S protein itself (chain E), the IFD results revealed that compounds with CIDs 130345966, 5315615, 439589, 131676018, and 162859052 demonstrated the strongest binding affinities, ranging from −14.8 to −13.5 kcal mol^−1^ (Table S3).

Regarding the second binding site, located on ACE2 (chain A), the top hit compounds had CIDs of 133561492, 74539584, 390474, and 159600901, with binding affinities ranging from −11.0 to −9.5 kcal mol^−1^ (Table S3). The IFD results for the third binding site showed slightly lower binding affinity values, with −10.8 kcal mol^−1^, with the top hit compounds having CIDs of 125990, 74336856, 51352598, and 142750760 (Table S3). Overall, these findings suggest that the identified compounds have promising binding affinities to the Mpro enzyme and different binding sites of the S protein, including the ACE2 receptor and the S protein itself.

**Figure 12:**
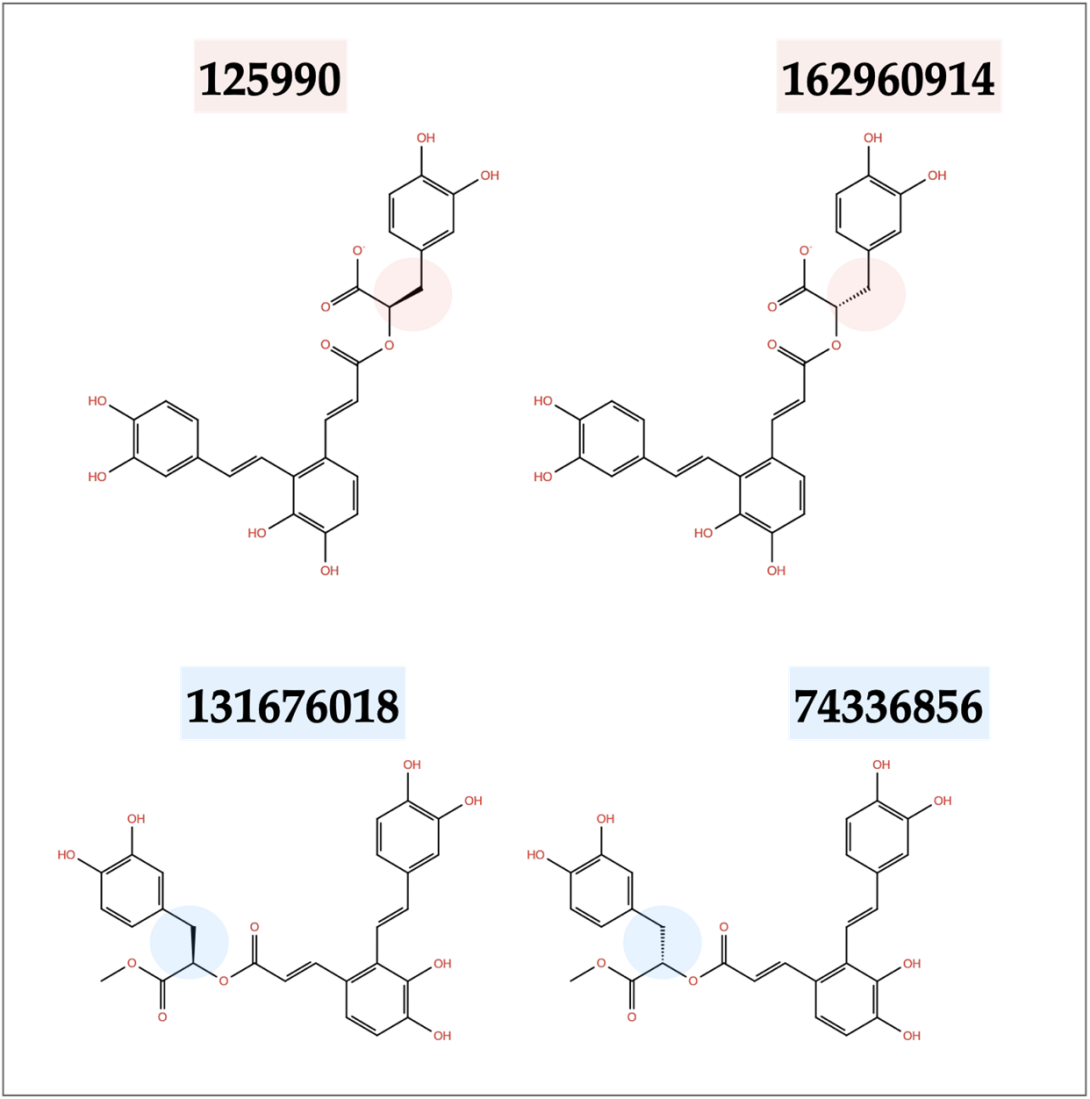
The 2D structures of the two multi-target inhibitors for SARS-CoV-2 and their enantiomers.

**Figure 13:**
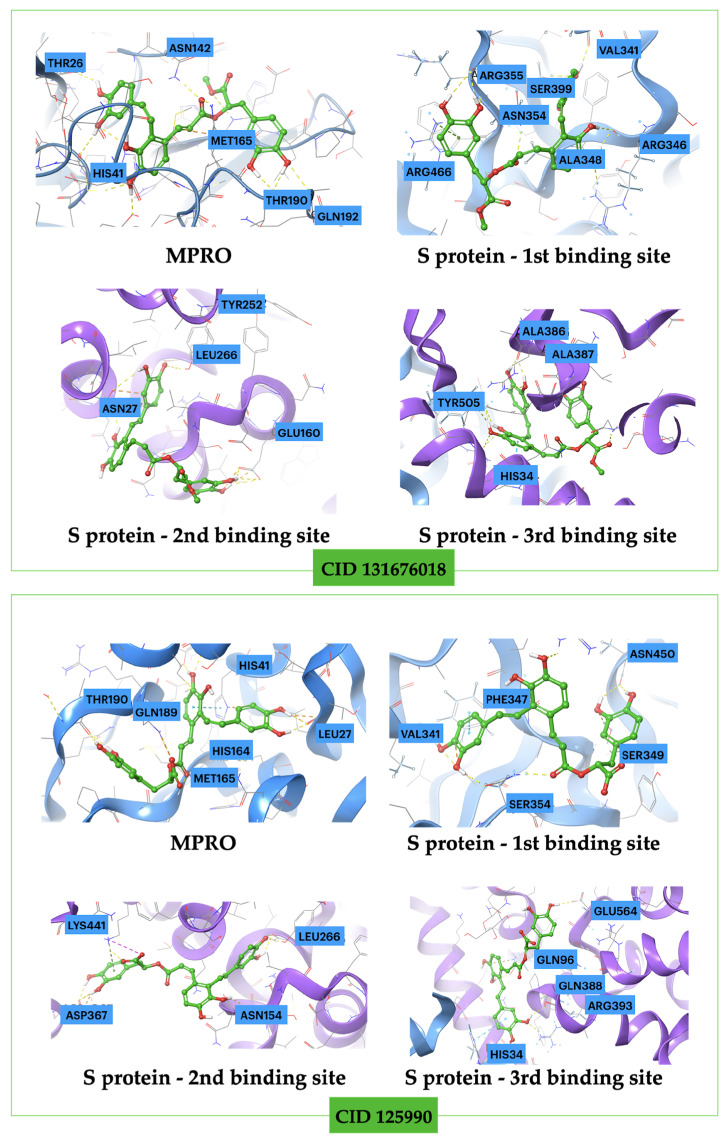
Visualization of the three-dimensional intermolecular interactions within protein–131676018 and protein–125990 complexes, conducted for each binding site following the completion of IFD studies. The carbon atoms of the two ligands are represented in green, and the oxygen atoms in red. Hydrogen bonds are represented by yellow dashed lines, while pi–cation interactions are depicted in green.

Furthermore, through IFD studies, we successfully discerned two compounds and their corresponding enantiomers that consistently exhibited robust binding affinities across all the examined binding sites. Specifically, the enantiomeric pairs 131676018/74336856, and 162960914/125990 (as illustrated in Figure 12) all displayed binding affinities within the same range (as outlined in Table 2). In our subsequent analysis of the interactions between each ligand–Mpro and ligand–S protein complex, we noted the formation of numerous contacts (Figure 13). These interactions collectively elucidated the exceptional binding affinities observed in our study.

This highlights their promise as multi-target inhibitors against both SARS-CoV-2 Mpro and the S protein. These compounds not only exhibited robust binding affinities (as illustrated in Table 2) and formed interactions with crucial residues at each binding site but also possessed the remarkable capability to concurrently target and inhibit two pivotal proteins essential for the virus’s replication and entry processes. Their dual inhibitory action positions them as promising candidates for the prevention of COVID-19.

## 3. Discussion

The primary intent of our research endeavor was to identify novel inhibitors targeting two critical components of SARS-CoV-2: Mpro and the S protein. Given their paramount role in viral replication and host cell entry, discovering new inhibitors holds immense promise in combating the ongoing global pandemic. By employing state-of-the-art computational techniques, our aim was to uncover compounds that exhibit potent binding affinities and inhibit the functions of both Mpro and the spike protein.

In this comprehensive in silico study, we employed a combination of molecular docking and molecular dynamics simulations to target these two protein components of SARS-CoV-2. Our overarching objective was to unearth potent inhibitors for combating COVID-19 from natural products and antihypertensives. Within this study, the IFD results unveiled four natural products, namely, salvianolic acid b, rosmarinic acid, curcumin, and quercetin, each exhibiting robust binding affinities. To delve deeper into the binding effects, we conducted MD simulations, which revealed that rosmarinic acid and quercetin demonstrated stable and persistent binding to Mpro. To substantiate these findings energetically, we performed MM-GBSA calculations, which consistently corroborated our previous analyses.

Turning our attention to the S protein, encompassing three distinct binding sites, including one on the S protein itself, another on ACE2, and a third at the interface of the S protein and ACE2, our IFD results identified salvianolic acid b, rosmarinic acid, and quercetin as high-affinity binders. Subsequent MD analysis showcased that salvianolic acid b and rosmarinic acid formed robust and enduring bonds. These results were further affirmed by MM-GBSA calculations.

Motivated by these promising outcomes, we conducted a similarity search using the parent compounds salvianolic acid, rosmarinic acid, and quercetin to identify structurally related compounds. This search yielded a total of 115 potential structures. Finally, through IFD studies on Mpro (featuring a single binding site) and the S protein (featuring three binding sites), we identified two salvianolic acid analogues, displaying favorable binding affinities for both Mpro and the S protein simultaneously, capable of acting as multi-target inhibitors. This research represents a significant stride toward the development of effective therapeutic strategies against COVID-19. While our computational findings are indeed promising, it is crucial to emphasize the necessity of further validation through in vitro and in vivo studies.

## 4. Materials and Methods

### 4.1. Protein Structure Preparation

The protein structures were prepared using the Protein Preparation Wizard available in Schrödinger Suites [47]. Crystal structures of the SARS-CoV-2 Mpro enzyme and S protein, available in the Protein Data Bank (PDB) with PDB IDs 6LU7 and 6M0J, respectively, were used [12,48]. This procedure involved adding missing hydrogens, filling in missing side chains and loops using Prime [49], and assigning partial charges using the OPLS_2005 force field [50]. A minimization procedure was executed, during which the movement of heavy atoms can be constrained. This enabled the alleviation of structural strain, while ensuring that the ultimate outcome remained relatively close to the initial input geometry. Notably, hydrogen atoms remained unrestrained throughout this process, facilitating the fine-tuning of the optimized hydrogen-bonding network achieved in the preceding step. Additionally, the hydrogen bonding structure was improved, and geometry optimization was carried out, which were all performed using the Protein Preparation Wizard [47]. This process ensured that the protein structures were appropriately prepared for docking, allowing for accurate predictions of ligand binding interactions.

### 4.2. Ligand Structure Preparation

The ligands’ structures (Figure 1) were designed and further subjected to LigPrep algorithm available in Schrödinger Suites to maintain the specific stereochemistry of each molecule [51]. Proper treatment of the protonation states of the compounds at physiological pH (~7.4) was also carried out using Hammett and Taft methods, along with an ionization tool, to generate chemically sensible 3D models. Furthermore, MacroModel program was used to optimize the geometries and perform conformational search of the ligands’ structures while ensuring that the chiral centers retained their proper chiralities. The resulting conformations of the ligands were further minimized with MacroModel, using OPLS_2005 as force field [50] and implicit water solvent, while the minimization step was performed via the Polak–Ribiere conjugate gradient (PRCG) method with a threshold of 0.01 kcal mol^−1^.

### 4.3. Molecular Docking Studies

Molecular docking studies were performed by means of Schrödinger’s Glide/XP 2017-1 software [52], utilizing the Induced Fit Docking (IFD) method, which considers the flexibility of both the ligand and protein, thereby allowing conformational changes to more closely match one another and improving predictions of the binding mode by producing more biologically relevant outcomes [53]. This is in contrast to rigid docking techniques, where the protein’s atomic positions are fixed, and only the ligand torsional degrees of freedom are allowed to change during docking. Docking calculations were performed by using two boxes: an outer box that specified the volume in which the grid potentials are computed and an inner box that specified the volume that the ligand center searches in. For the protein structures considered, the outer grid box dimensions were set to 26 × 26 × 26 Å^3^, while the inner box was set to the default size (i.e., 10 × 10 × 10 Å^3^) [52] for the definition of each of the considered binding sites.

The binding sites we focused on in this study had specific amino acid residues that played crucial roles in molecular interactions. For SARS-CoV-2 Mpro, the binding site was characterized by several key residues, including His41, Met49, Asn142, Met165, Glu166, Leu167, Pro168, Phe185, Gln189, and Ala191 [21].

Regarding the S protein (S protein) complexed with angiotensin-converting enzyme 2 (ACE2), we conducted molecular docking studies under three distinct scenarios, each with its own designated binding site. In the first scenario, we modified the binding area and focused on the S protein itself (chain E), specifically targeting residues Phe338, Glu340, Val341, Phe342, Asn343, Arg346, Tyr351, Arg355, and Asp364 [54]. In the second scenario, we identified a binding site within ACE2 (chain A), defined by specific residues Met152, Ala153, Asn154, Ser155, Leu156, Arg161, Leu248, Tyr252, Leu266, Gly268, Asn277, Leu278, Tyr279, Ser280, and Leu281.

Lastly, the third binding site is located at the intersection of the S protein and ACE2, which we referred to as the “new binding site”. This site was precisely defined by the residues Lys26, Asp30, Val93, Pro389, Arg408, His417, Phe555, Asn556, and Arg559, as proposed by Durdagi et al. [21].

During glide docking calculations, the OPLS_2005 force field was used, and the preparation constrained refinement was applied [50]. Automatic trimming of side chains based on B factors and refinement of the protein side chains using Prime was also performed [49]. Subsequently, the extra precision predicted binding score (XP GScore) was calculated for each ligand, and the XP GScore scoring function took into account various factors, such as bond type and electrostatic, van der Waals, and hydrophobic interactions between the protein and ligand during complex formation. The “post docking minimization” option was enabled, keeping a maximum of 10 poses per ligand. The results of our induced molecular docking experiments for each scenario are provided with the Supplementary Materials.

### 4.4. Molecular Dynamics Simulations

MD simulations were conducted to assess the stability of the complexes formed between proteins and natural products or antihypertensive drugs, which were initially identified through molecular docking calculations. These simulations aimed to characterize the key interactions responsible for the formation of “protein-ligand” complexes in each chemical system under investigation. Additionally, simulations were performed for the apo proteins, Mpro and S protein bound to ACE2.

To achieve this, the most energetically favored pose of the “protein-ligand” complexes derived from docking studies was selected as initial pose for the MD simulations. OPLS_2005 force field was used in order to describe the intermolecular forces governing the formation of the complexes [50,55], while long-range electrostatics were treated with the particle mesh Ewald method (PME) with grid spacing of 0.8 Å. Van der Waals and short-range electrostatics were truncated at 9.0 Å. The OPLS_2005 force field stands as an advanced iteration of the OPLS-AA force field, tailored to accommodate the diverse functional groups inherent to organic molecules. Notably, the force field’s enhancements encompass the meticulous retrofitting of rotational parameters, meticulously calibrated to replicate the conformational energies obtained from high-precision quantum computations. Moreover, the integration of supplementary charge parameters addresses the full spectrum of organic functional groups. In the context of protein molecules, parameter optimization further refines accuracy, culminating in an all-encompassing force field with the capacity to yield precise and reliable calculations for a wide range of molecular systems. In order to prepare the system for the MD simulations, the “protein-ligand” complexes were fully hydrated with explicit solvent Transferable Intermolecular Potential 3-Point (TIP3P) [56] modeled water molecules using cubic unit cells and applying periodic boundary conditions to all directions. The dimensions of the simulation box were 10^3^ Å, and the electro neutrality of the total system charge was achieved by adding Na^+^ ions. A stable salt concentration (c = 0.15 M/cellular concentration) was reached by including additional Na^+^ and Cl^−^ ions to the chemical system. The solvated system was initially minimized using a hybrid method of steepest decent and limited-memory Broyden–Fletcher–Goldfarb–Shanno (LBFGS) algorithms, while following heating and equilibration processes were applied by the default relaxation protocol provided by Desmond simulation package [57].

In particular, the default Desmond protocol includes five relaxation steps. Firstly, the system is heated in the NVT ensemble using Brownian dynamics at ~10 K with small time steps and then a 12 ps simulation in the canonical ensemble is performed with the use of Berendsen thermostat at the same temperature. The solute non-hydrogen atoms is restrained in both steps.

Furthermore, the next step of the protocol involves a 12 ps simulation in the NPT ensemble using Berendsen thermostat and barostat in order maintain a constant temperature and pressure constant at 10 K and 1 atm, respectively. Finally, the last two relaxation steps of the NPT ensemble include a 12 ps simulation with the Berendsen thermostat and barostat to reach the target temperature of 310 K (body temperature), while simultaneously maintaining stable pressure at 1 atm. The solute non-hydrogen atoms are restrained in all the aforementioned relaxation steps, except for the last step, which includes a 24 ps simulation in NPT ensemble (Berendsen thermostat and barostat), where no restraints are applied to the chemical system [58].

The equations of motion are integrated using the multistep RESPA integrator with an inner time step of 2 fs for bonded interactions and non-bonded interactions within a cutoff of 9.0 Å. An outer time step of 6.0 fs is used for non-bonded interactions beyond the cut-off.

Finally, production runs of 200 ns are conducted for all the equilibrated systems at the same temperature and pressure conditions, using the GPU implementation of Desmond, which allows for faster computation of the simulation by providing an adequate sample size for analyzing the molecule’s binding mode to the protein’s cavity [57].

Furthermore, in order to discover the most frequent conformational state visited by the “protein-ligand” complexes during the duration of the simulations, as well as to quantitatively assess the MD simulations’ results, we employed the Desmond Trajectory Clustering tool implemented in Maestro suite [57]. Clustering enables the identification of distinct conformational states of a biomolecule during MD simulations, and it leads to the discovery of statistically representative structures of the chemical system (i.e., cluster centroids), facilitating subsequent analysis. Clustering was performed by setting a frequency value of 20 for grouping the protein–ligand conformations into 10 clusters.

Trajectory analysis was performed by utilizing Desmond analysis tools. Structural analysis involved the evaluation of the mass-weighted root-mean-square deviation (RMSD) of the protein backbone Cα atoms and the RMSD of the ligands aligned to ligand with respect to the initial system coordinates (“protein-ligand” pose derived by docking studies) [59]. RMSD of the protein backbone Cα atoms was used as a measure of system convergence. In addition, root-mean-square fluctuation (RMSF) of the protein backbone Cα atoms was employed as an indicator of local structural flexibility throughout the trajectory analysis, providing insights into dynamic changes within the system [57].

Furthermore, supplementary hydrogen bond analysis was applied in order to discover the key H-bond interactions that govern the ligands’ binding to the protein’s active site. All the other intermolecular interactions (salt bridges, pi–cation interactions, and pi–pi interactions) that are formed between the protein and the ligand in every complex werer quantitatively evaluated by the Desmond Trajectory analysis tool [57].

### 4.5. MM-GBSA Calculations

Binding free energies were calculated using the prime molecular mechanics/generalized Born surface area (MM-GBSA) method, using the VSGB solvation model [60]. This approach was used to study the thermodynamics of “protein-ligand” interactions and to aid in drug discovery efforts by predicting the binding affinity of potential drug candidates [59]. The relevant equations of the VSGB method can be found elsewhere [60,61,62]. Briefly, the MM-GBSA method calculates the free energy of binding ($\Delta G_{bind}$) between a receptor and a ligand for every selected trajectory frame as

(1) $$\Delta G_{bind}=G_{complex}-(G_{receptor}+G_{ligand})$$

where, $G_{complex}$, $G_{receptor}$, and $G_{ligand}$ denote the absolute free energy of the complex, receptor, and ligand, respectively. The method is based on the idea that a ligand to receptor binding free energy can be accurately rendered by combining molecular mechanics ($\Delta E_{MM}$, which includes the sum of internal energy change, $\Delta E_{int}$, van der Waals, $\Delta E_{vdW}$, and electrostatic energy, $\Delta E_{elec}$, contributions) and polar ($\Delta G_{solv,\;p})$ and nonpolar ($\Delta G_{solv,\;np})$ solvation components [59].

(2) $$\Delta G_{bind}=\Delta E_{MM}+\Delta G_{solv,p}+\Delta G_{solv,np}$$

Prime-MM-GBSA evaluates the bonded and non-bonded contributions from the OPLS-AA protein force field using a variety of physics-based correction terms, including terms for improved handling of pi-stacking and hydrogen bonding interactions [50,56]. The implicit solvent model is based on the variable dielectric surface generalized Born (VD-SGB) method, where the variable dielectric value for each residue is fitted to a significant number of side-chain and loop predictions. A parametrized hydrophobic term is used to determine the nonpolar solvation free energy. It is important to note that the entropy contribution to the free energy was not calculated in this analysis; therefore, the presented results are binding enthalpies. To compute the binding energetics, the thermal_mmgbsa.py python script embedded in the Schrödinger suite was employed for the final converged 40 ns of the simulation (i.e., 200 frames), utilizing the default sampling method (i.e., minimize flexible protein residues) [49].

### 4.6. Similarity Search

Enalos Suite is a cheminformatics platform for drug discovery developed by NovaMechanics Ltd., Nicosia, Cyprus, which allows for the use of robust models with high predictive power according to OECD principles. The “Similar Compounds” function available in Enalos Suite was used for obtaining the compounds included in the PubChem database that are similar to the three initial query compounds: salvianolic acid, rosmarinic acid and quercetin. The Tanimoto coefficient, a popular similarity measure, was used to identify how similar the molecules are in terms of chemical structure. This metric calculates the number of chemical features that are common in two molecules compared to the number of features that can be found in wither. By tuning the percentage of desirable similarity to 99%, a total of 115 similar compounds were identified for salvianolic acid, rosmarinic acid, and quercetin [63,64].

## 5. Conclusions

The present study identified two potential analogues of salvianolic acid from a comprehensive dataset encompassing natural products and hypertensives, employing various computational tools. In order to gain deeper structural insights into their binding effects on both Mpro and the S protein, we conducted molecular docking and molecular dynamics simulations. Extensive structural analysis and free energy calculations with the MM-GBSA method demonstrated the stability of each considered ligand–protein complex. Furthermore, based on a similarity search and subsequent IFD studies within the obtained dataset, we proposed two novel compounds, namely, (2*R*)-3-(3,4-dihydroxyphenyl)-2-[3-[2-[2-(3,4-dihydroxyphenyl)ethenyl]-3,4-dihydroxyphenyl]prop-2-enoyloxy]propanoic acid (CID 125990) and methyl 3-(3,4-dihydroxyphenyl)-2-[3-[2-[2-(3,4-dihydroxyphenyl)ethenyl]-3,4-dihydroxyphenyl]prop-2-enoyloxy]propanoate (CID 74336856), as promising multi-target inhibitors capable of targeting both SARS-CoV-2 Mpro and the S protein. In conclusion, this study identified two potential lead compounds that show promise for treating COVID-19. The compounds discovered by our thorough computational investigation are ideal candidates for subsequent in vitro and in vivo validations.

## Figures and Tables

**Table 1:** Total free energy values calculations of the “protein-ligand” complexes at each binding site through MM-GBSA.

Ligand	ΔG (kcal mol^−1^)			
	Mpro	S Protein 1st Binding Site	S Protein 2nd Binding Site	S Protein 3rd Binding Site
Curcumin	−47.5 ± 4.0	–	–	–
Quercetin	−33.8 ± 5.2	–	−41.0 ± 6.1	–
Rosmarinic acid	−45.2 ± 3.6	−41.2 ± 4.4	−41.0 ± 3.8	−40.4 ± 9.1
Salvianolic acid b	–	−56.5 ± 4.9	−42.6 ± 5.8	−35.3 ± 5.1

**Table 2:** Calculated binding affinities (in kcal mol^−1^) via IFD of selected compounds identified from similarity search to Mpro and S protein binding sites as multi-target inhibitors.

Ligand CID	Mpro	Binding S Protein 1st Binding Site	Affinity (kcal mol^−1^) S Protein 2nd Binding Site	S Protein 3rd Binding Site
131676018	−16.5	−13.7	−8.2	−8.9
74336856	−13.7	−10.5	−9.6	−10.9
162960914	−15.4	−10.6	−9.0	−9.7
125990	−15.1	−12.8	−9.0	−10.7

## Supplementary Materials

The following supporting information can be downloaded at https://www.mdpi.com/article/10.3390/ijms242115894/s1.
