# Supplementary material for: Exploring the Binding Effects of Natural Products and Antihypertensive Drugs on SARS-CoV-2: An In Silico Investigation of Main Protease and Spike Protein
Source: Int J Mol Sci. 2023 Nov 2;24(21):15894. doi: 10.3390/ijms242115894 (PMC10649947; doi:10.3390/ijms242115894)
Supplement: Supplementary file 1 [file ijms-24-15894-s001.zip › ijms-2641327-supplementary.pdf]

Exploring the binding effects of Natural products and Anti-hypertensives drugs on SARS-CoV-2: An *in silico* investigation of Main Protease and Spike Protein

Kalliopi Moschovou [1], Maria Antoniou [2,3], Eleni Chontzopoulou [1], Konstantinos D. Papavasileiou [2,3], Georgia Melagraki [4,*], Antreas Afantitis [2,3] and Thomas Mavromoustakos [1]

[1] Department of Chemistry, National and Kapodistrian University of Athens, 15771, Greece
[2] Department of ChemoInformatics, NovaMechanics Ltd., Nicosia 1046, Cyprus
[3] Department of ChemoInformatics, NovaMechanics MIKE., Piraeus, Greece
[3] Division of Physical Sciences & Applications, Hellenic Military Academy, 16672 Vari, Greece
* Correspondence: georgiamelagraki@gmail.com

Supplementary Material

Table S1. Binding affinities (kcal mol^{-1}) of the compounds considered at the Mpro binding site with the Induced Fit Docking method.

Compound	Binding affinity
**Cannabidiolic acid**	-5.9
**Cannabigerolic acid**	-5.4
**Caffeic acid**	-7.3
**Quercetin**	-7.8
**Curcumin**	-8.5
**Rosmarinic acid**	-9.5
**Salvianolic acid b**	n/d*
**Eprosartan**	-5.7
**Telmisartan**	n/d
**Irbesartan**	-2.9
**Candesartan**	-5.4
**Olmesartan**	n/d
**Valsartan**	n/d
**Azilsartan**	-6.7
**Losartan**	-6.7

*n/d: no docked

IFD Mpro - natural products

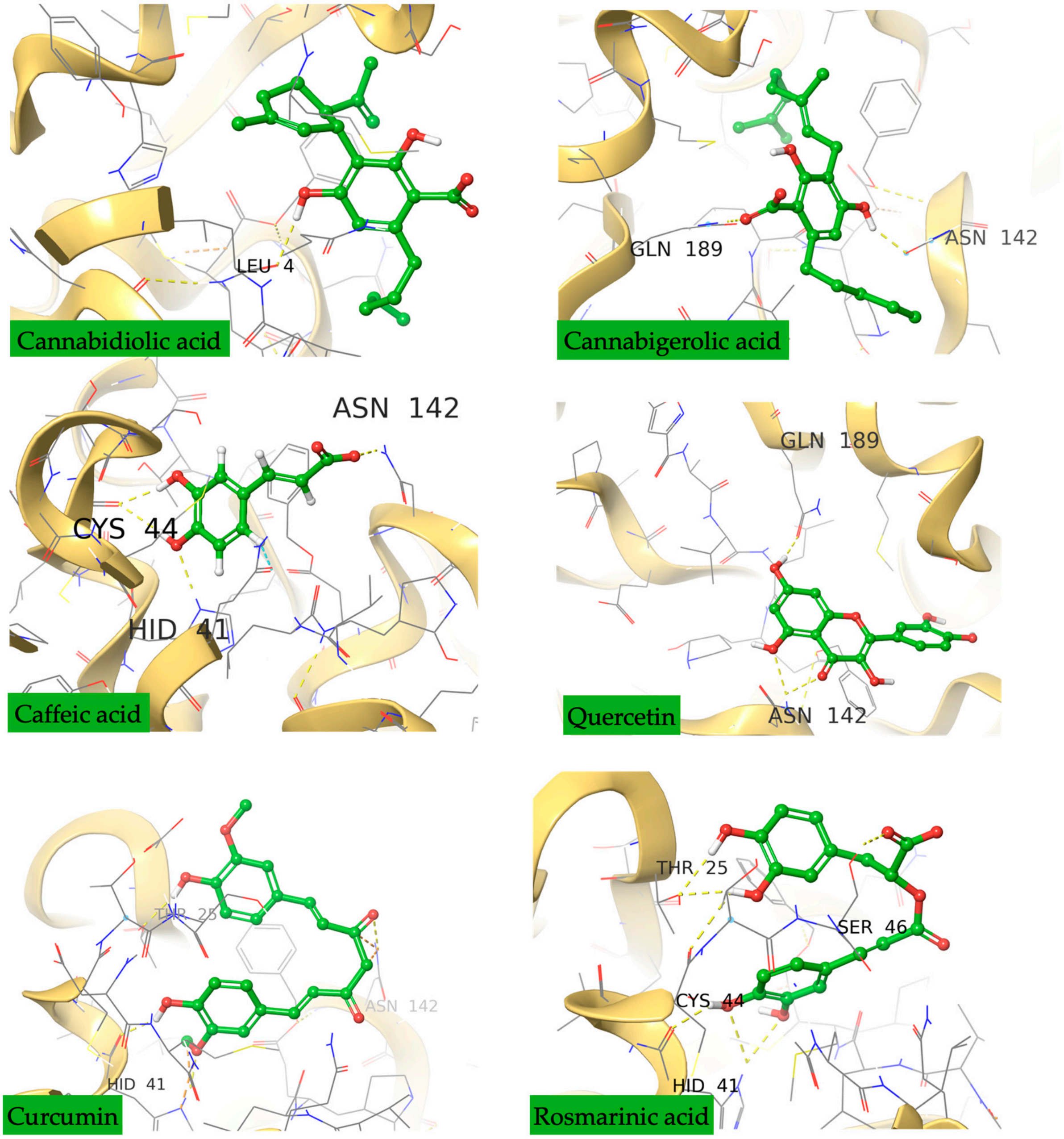

IFD Mpro - antihypertensives

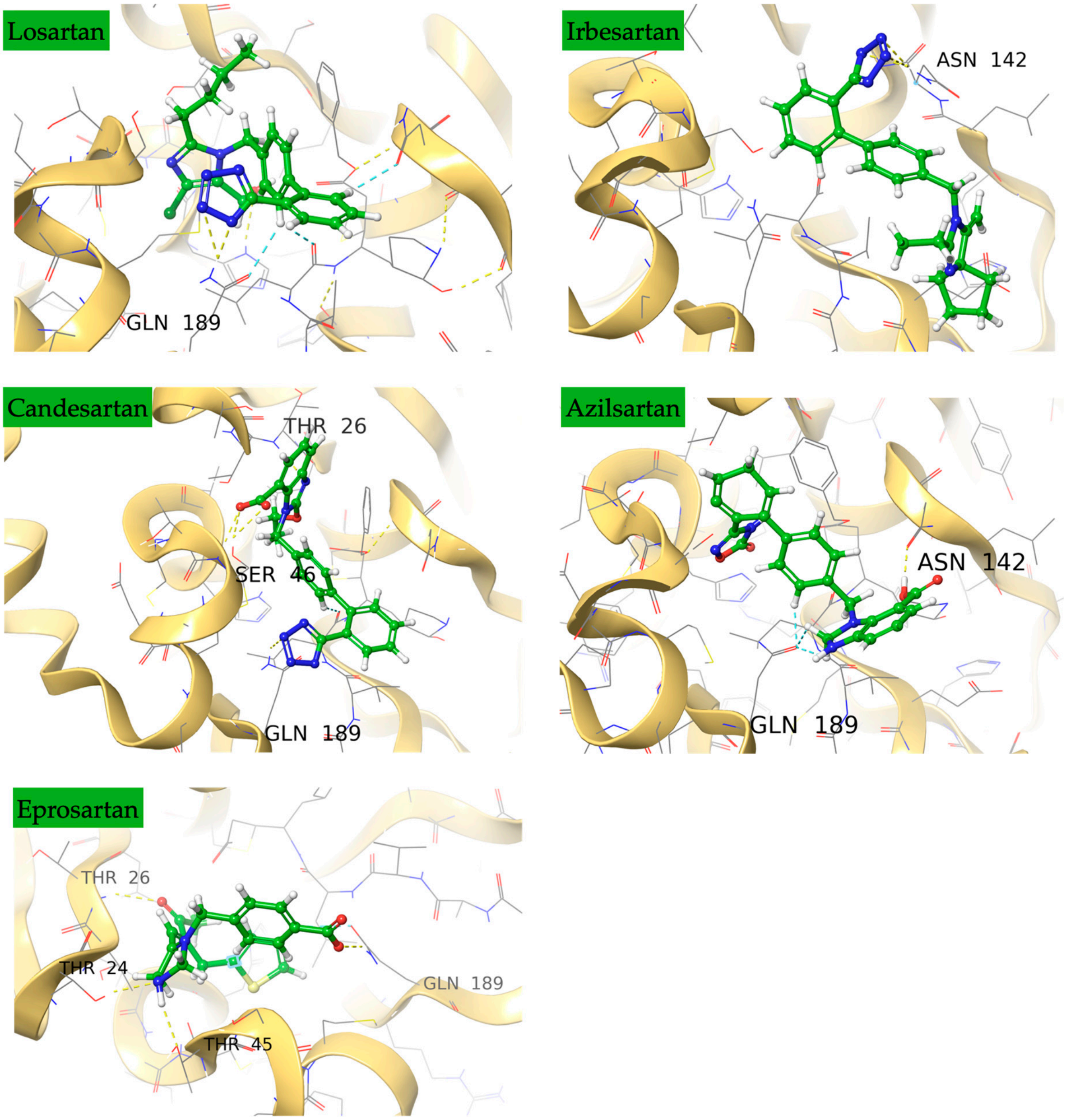

Figure S1. The docked poses of the "Mpro-natural products "and "Mpro-antihypertensives complexes" obtained from IFD. The Mpro is represented in yellow ribbon. Hydrogen bonds are represented by yellow dashed lines, aromatic hydrogen bonds by light blue dashes, while pi-cation interactions are depicted in green and salt bridges with magenta. The same color coding is consistently used for all figures presented.

Table S2. Induced Fit Docking (IFD) results for the three distinct binding sites of S protein PDB ID: 6M0J). Binding affinities are in units of kcal mol^{-1}.

Compound	**Binding Affinity Chain E (Spike)**	**Binding Affinity Chain A (ACE2)**	**Binding Affinity New binding site**
Cannabidiolic acid	-4.9	-7.7	-4.0
Cannabigerolic acid	-6.0	-6.9	-5.9
Caffeic acid	-7.1	-7.4	-4.7
Quercetin	-7.7	-10.4	-7.7
Curcumin	-7.3	-7.0	-6.6
Rosmarinic acid	-11.1	-9.9	-8.3
Salvianolic acid B	-14.2	-11.1	-10.0
Losartan	-6.0	-7.1	-5.1
Telmisartan	-3.8	-8.4	-3.6
Irbesartan	-3.3	-3.9	-2.8
Candesartan	-3.4	-3.6	-3.4
Olmesartan	-7.2	-4.8	-5.0
Valsartan	-4.2	-4.4	-3.0
Azilsartan	-6.7	-3.5	-5.4
Eprosartan	-4.9	-7.1	-6.4

IFD S protein - 1st binding site

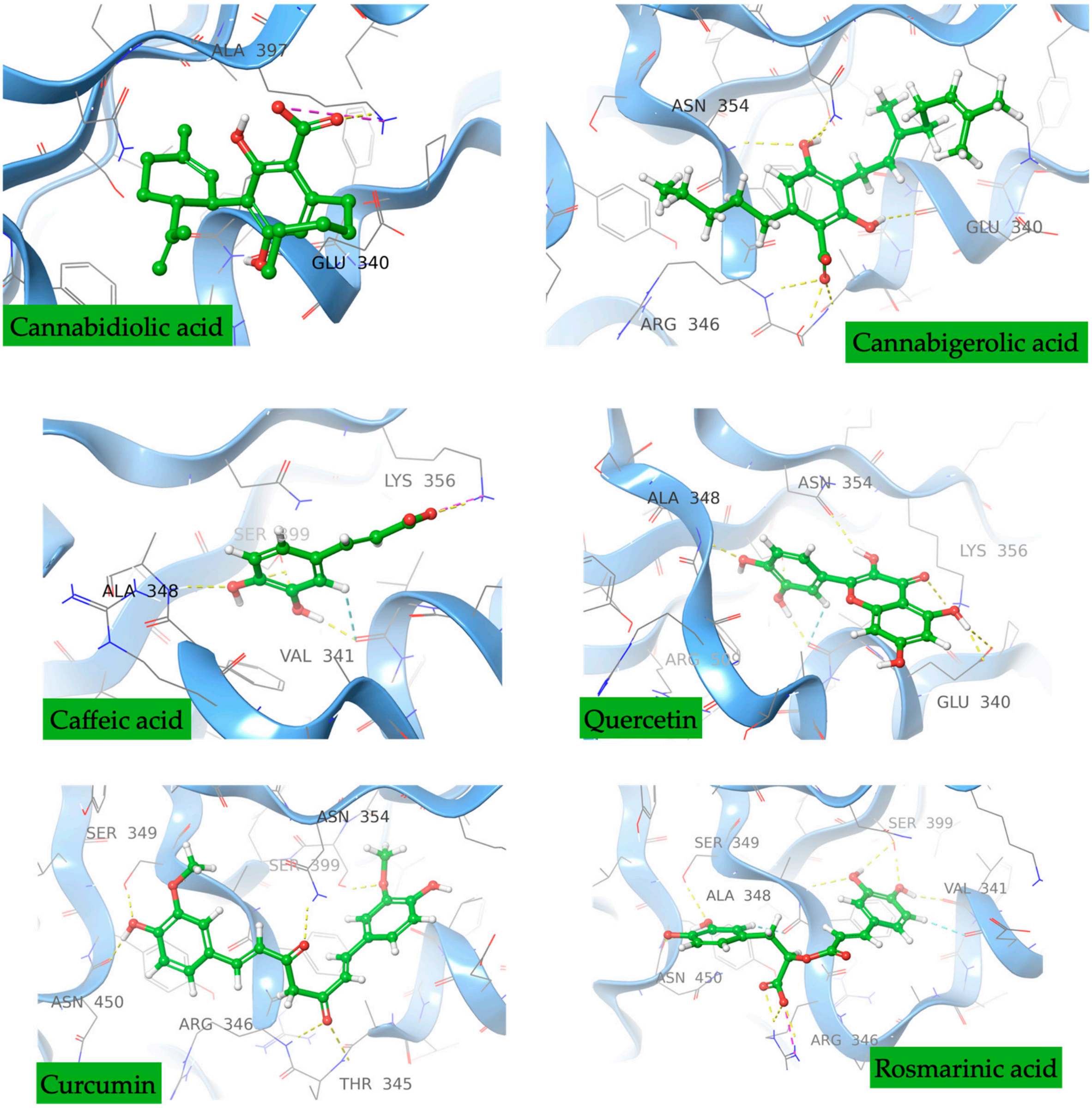

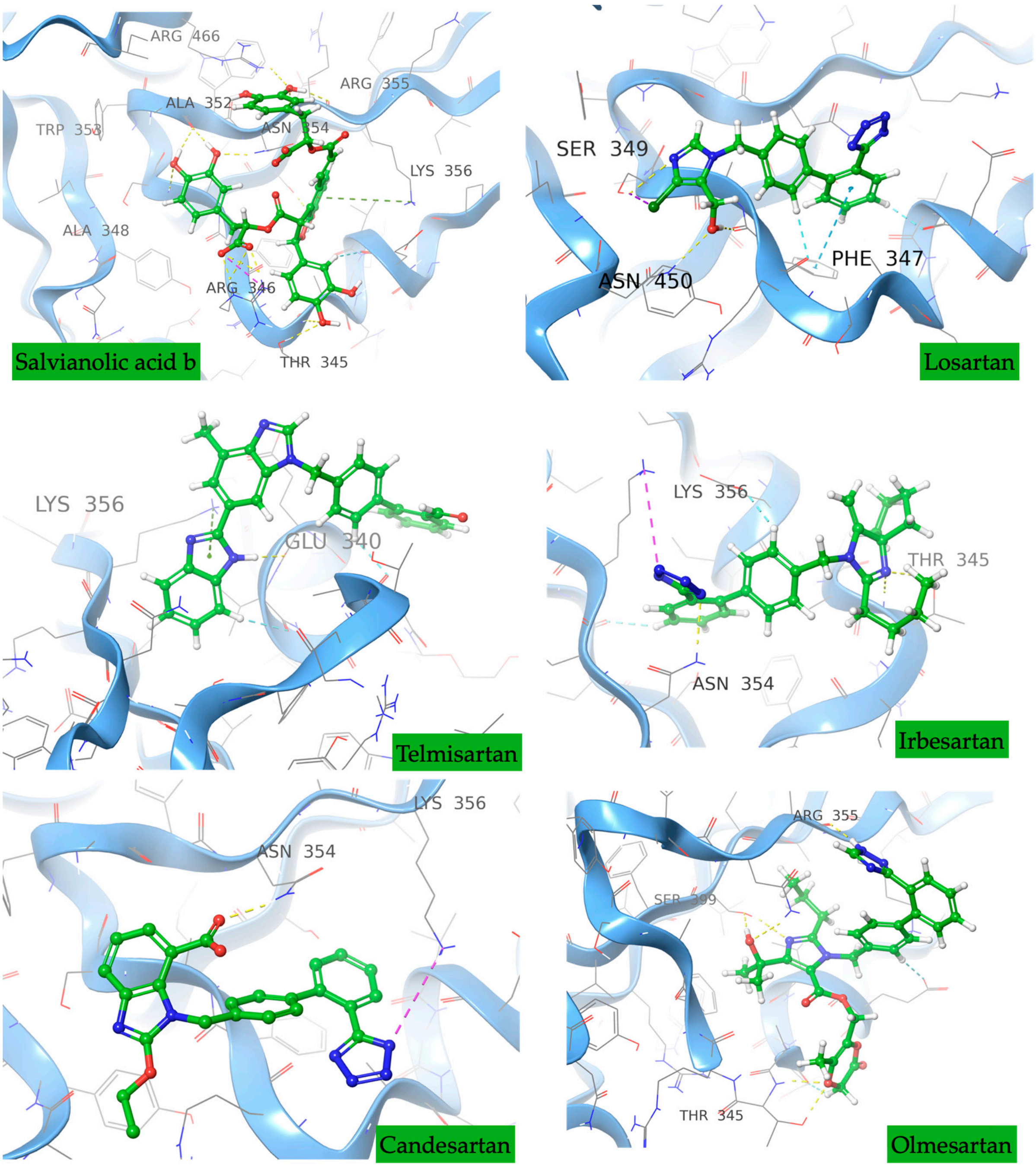
ARG 466
ARG 355
ALA 352
TRP 353
ASN 354
LYS 356
ALA 348
ARG 346
THR 345
Salvianolic acid b
SER 349
ASN 450
PHE 347
Losartan
LYS 356
GLU 340
Telmisartan
LYS 356
THR 345
ASN 354
Irbesartan
LYS 356
ASN 354
Candesartan
ARG 355
THR 345
Olmesartan

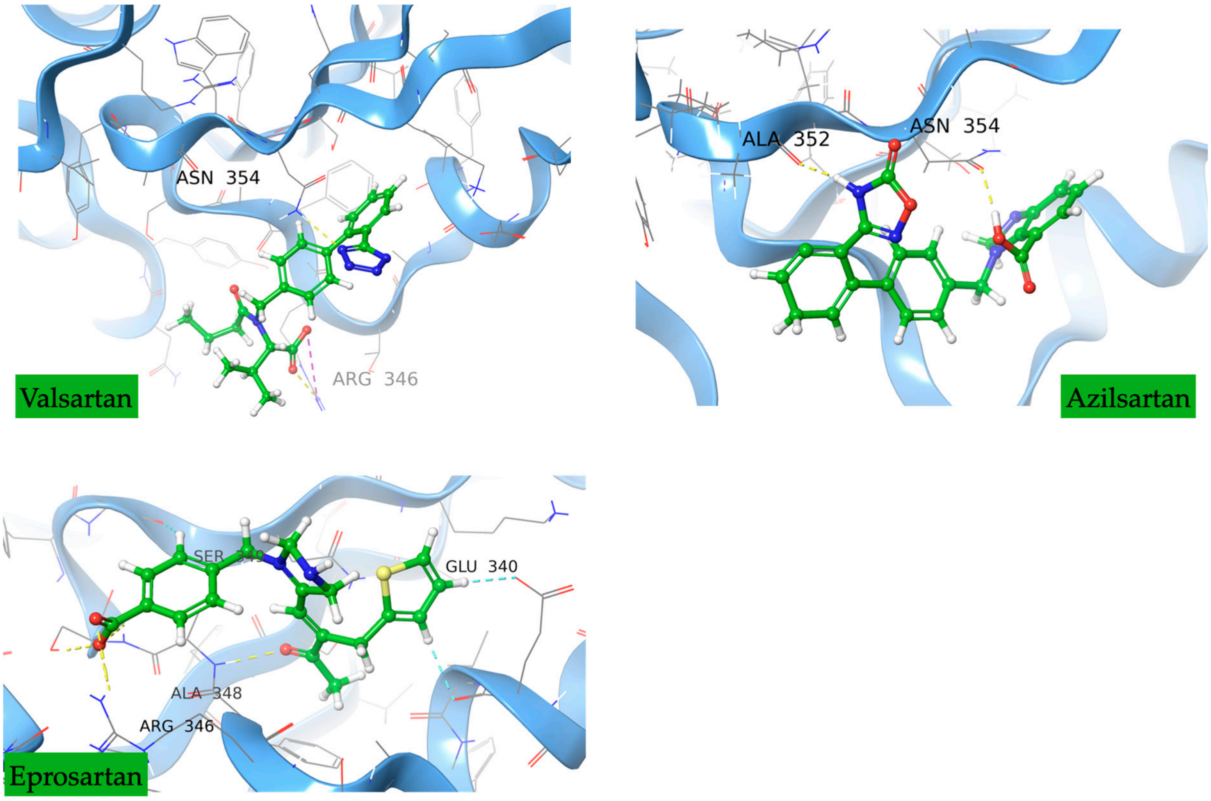

Figure S2. The docked poses of the “S protein-natural products” and “S protein -antihypertensives complexes” for the first binding site obtained from IFD. With light blue ribbon is represented the S protein (chain E) where the first binding site is located.

IFD S protein - 2nd binding site

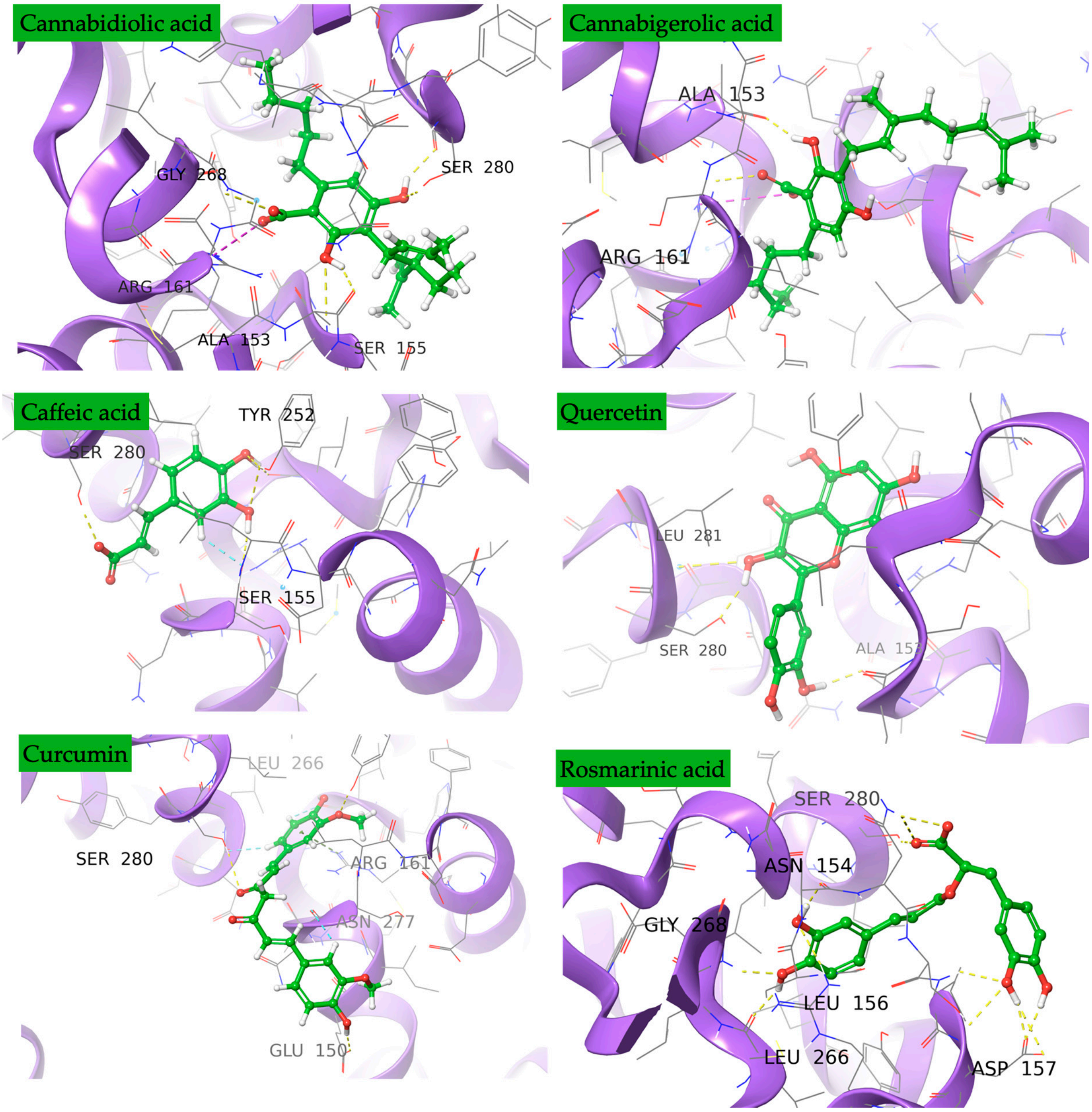

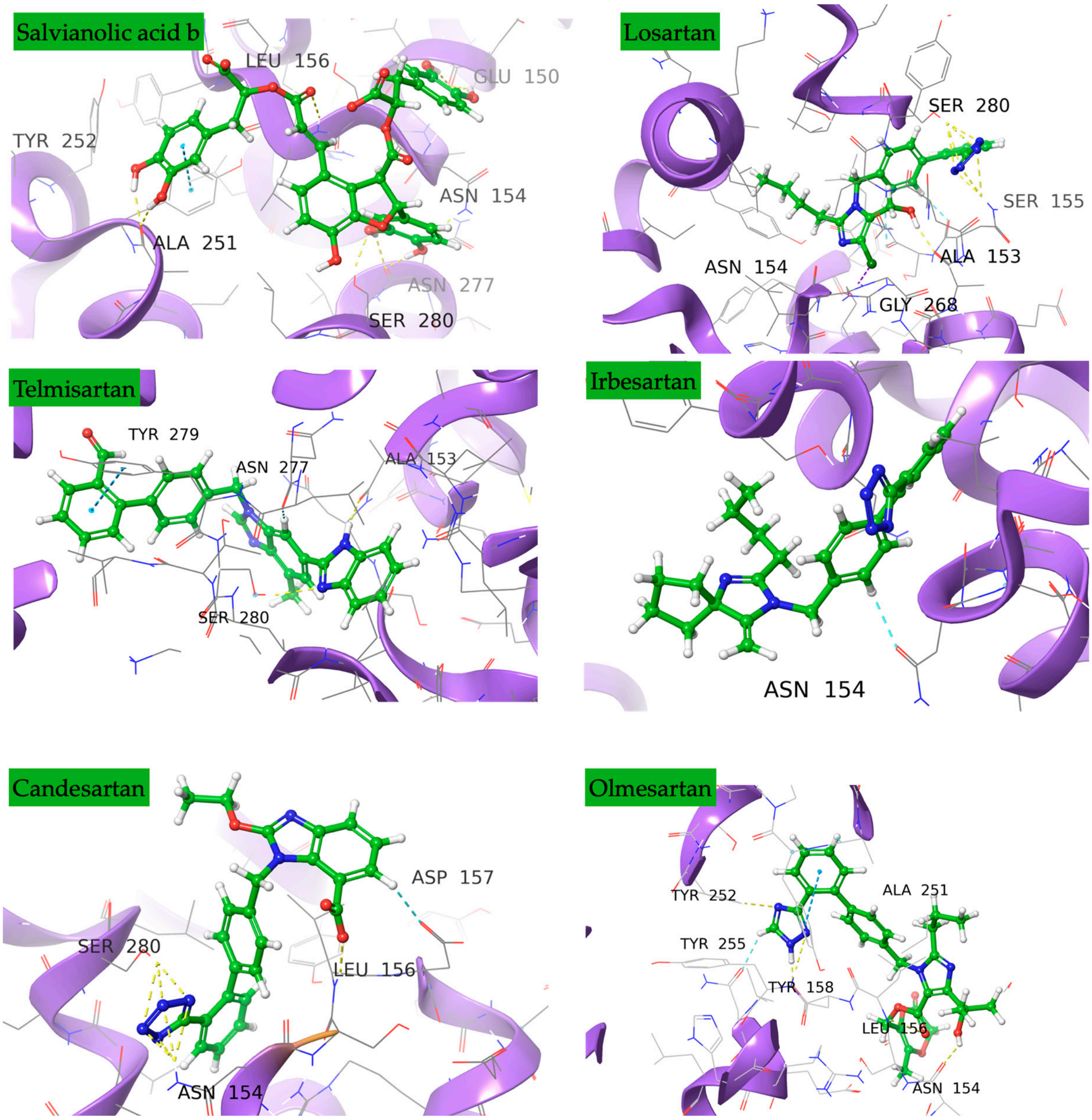
Salvianolic acid b
LEU 156
GLU 150
TYR 252
ASN 154
ALA 251
ASN 277
SER 280
Losartan
SER 280
SER 155
ALA 153
ASN 154
GLY 268
Telmisartan
TYR 279
ASN 277
ALA 153
SER 280
Irbesartan
ASN 154
Candesartan
ASP 157
SER 280
LEU 156
ASN 154
Olmesartan
TYR 252
ALA 251
TYR 255
TYR 158
LEU 156
ASN 154

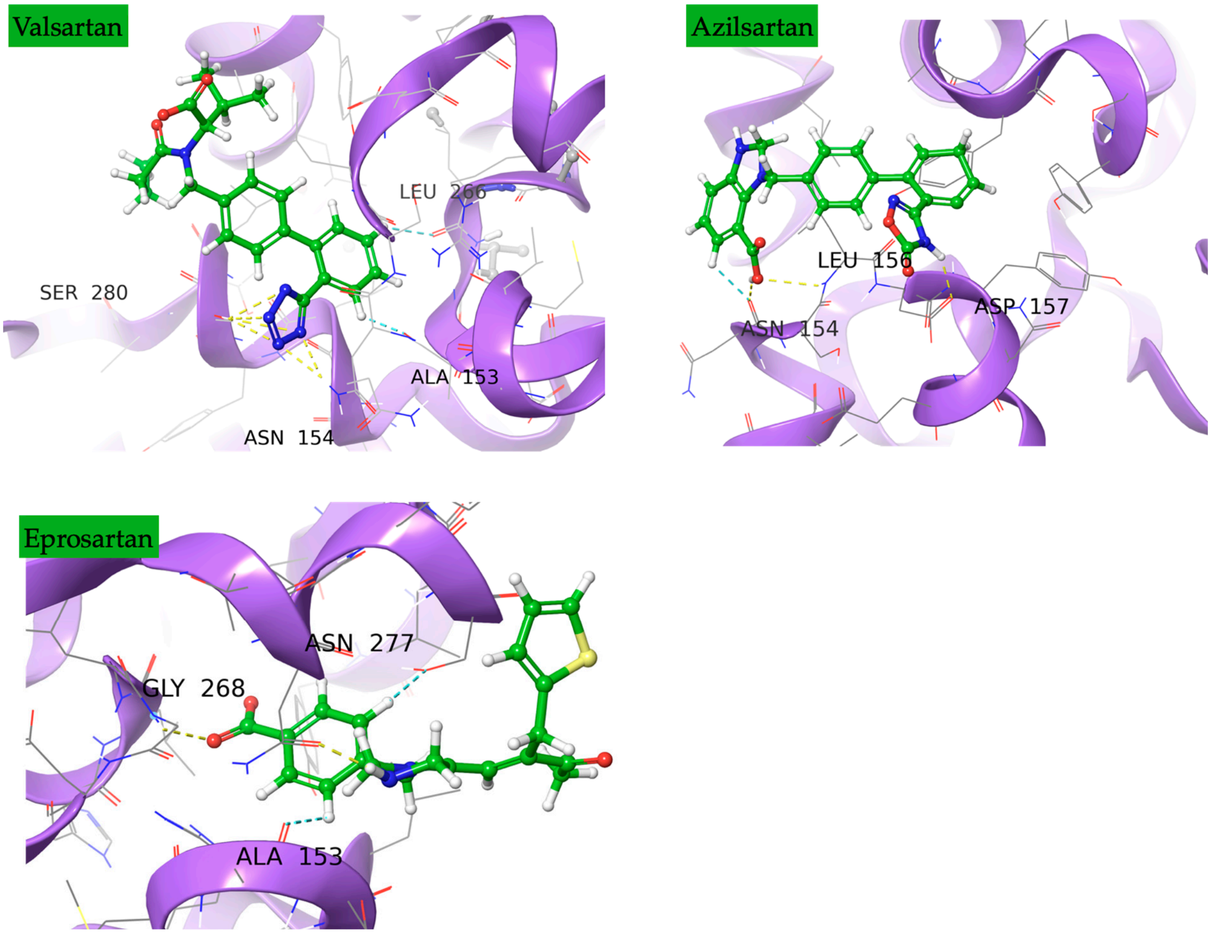

Figure S3. The docked poses of the complexes involving the S protein and natural products, as well as the S protein and antihypertensives, for the second binding site obtained from IFD. In the figure, the ACE2 (chain A), where the second binding site is located, is depicted with a purple ribbon.

IFD S protein - 3rd binding site

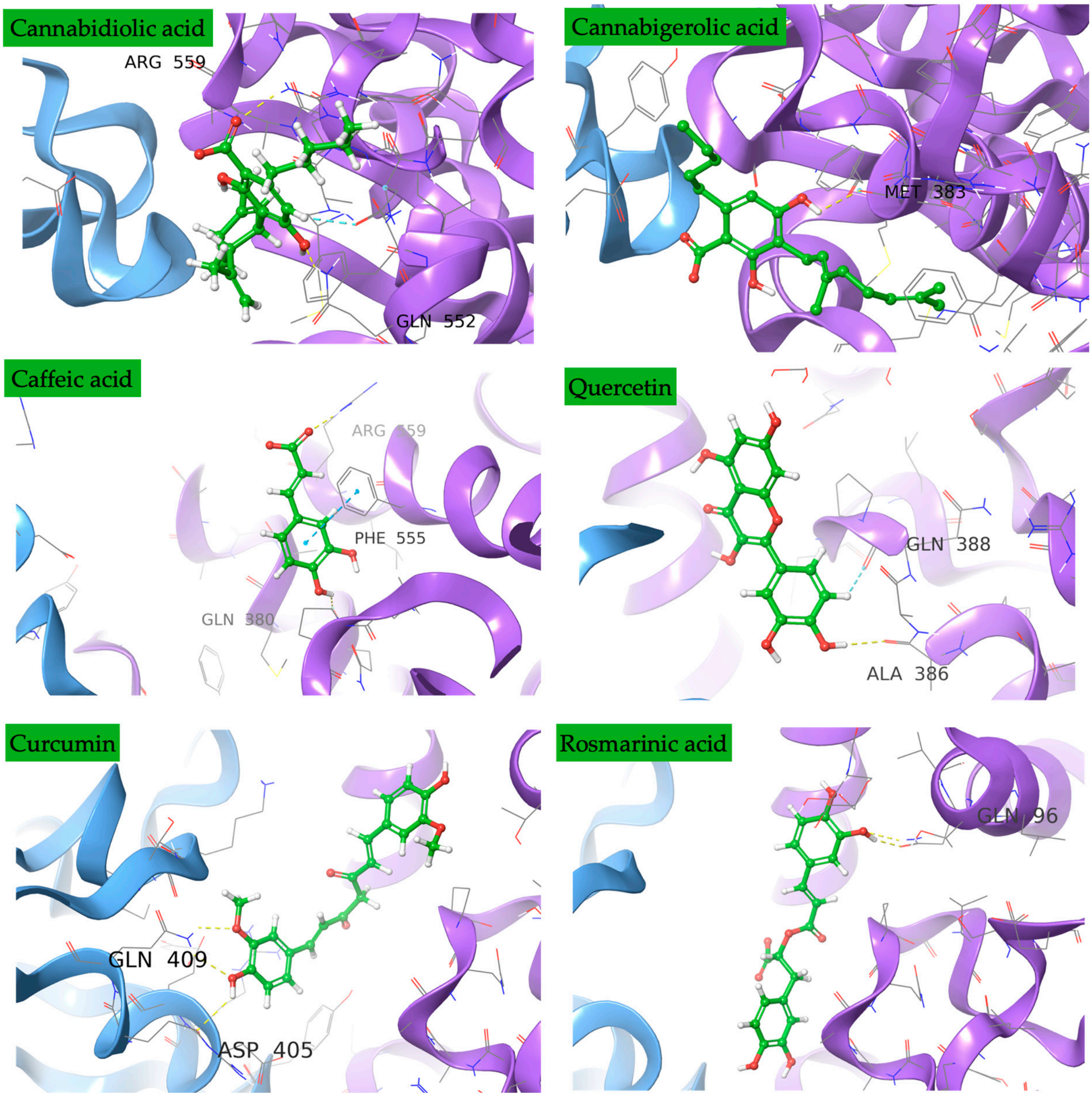

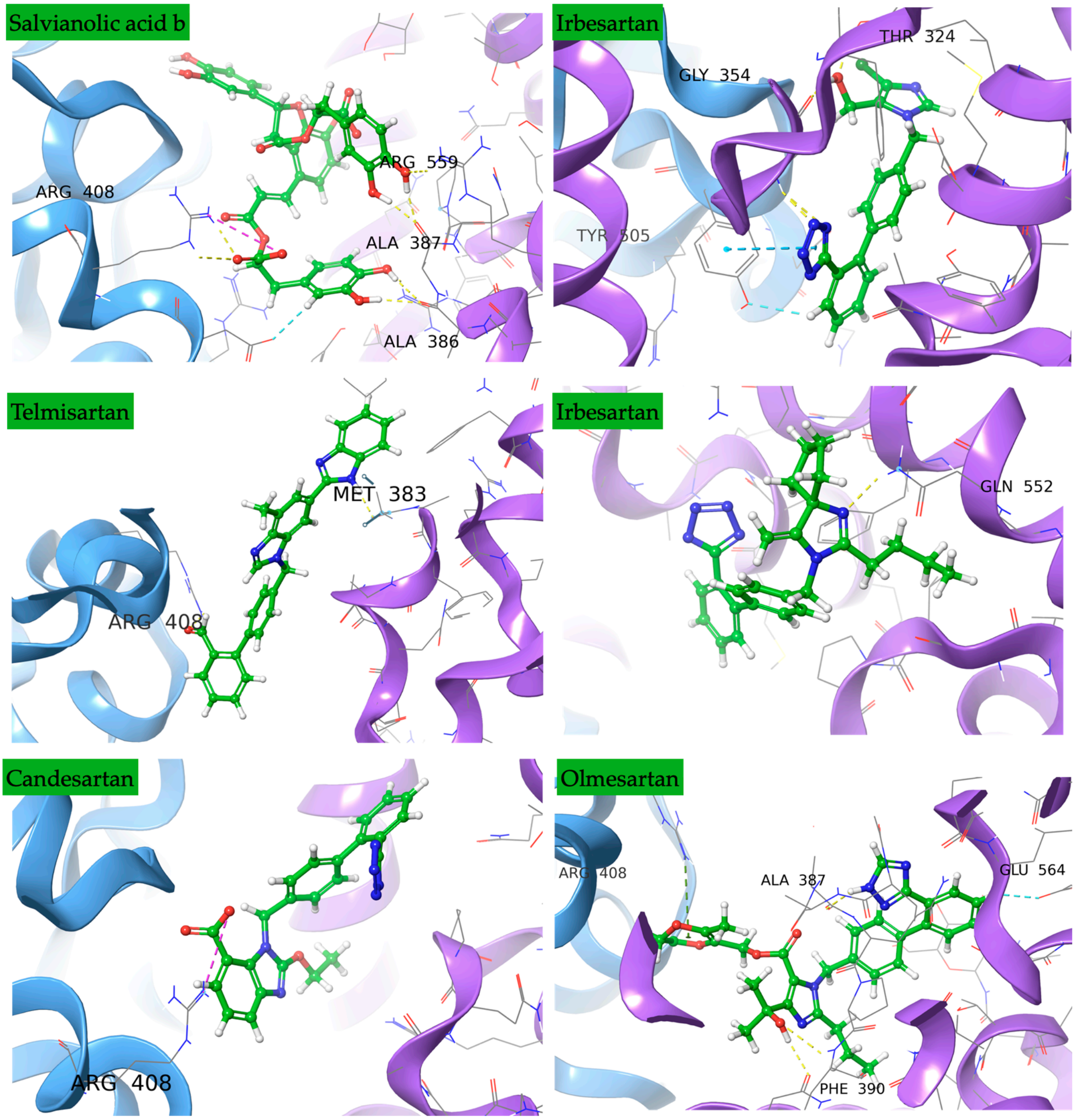

Salvianolic acid b
ARG 408
ARG 559
ALA 387
ALA 386
Irbesartan
GLY 354
THR 324
TYR 505
Telmisartan
MET 383
ARG 408
Irbesartan
GLN 552
Candesartan
ARG 408
Olmesartan
ARG 408
ALA 387
GLU 564
PHE 390

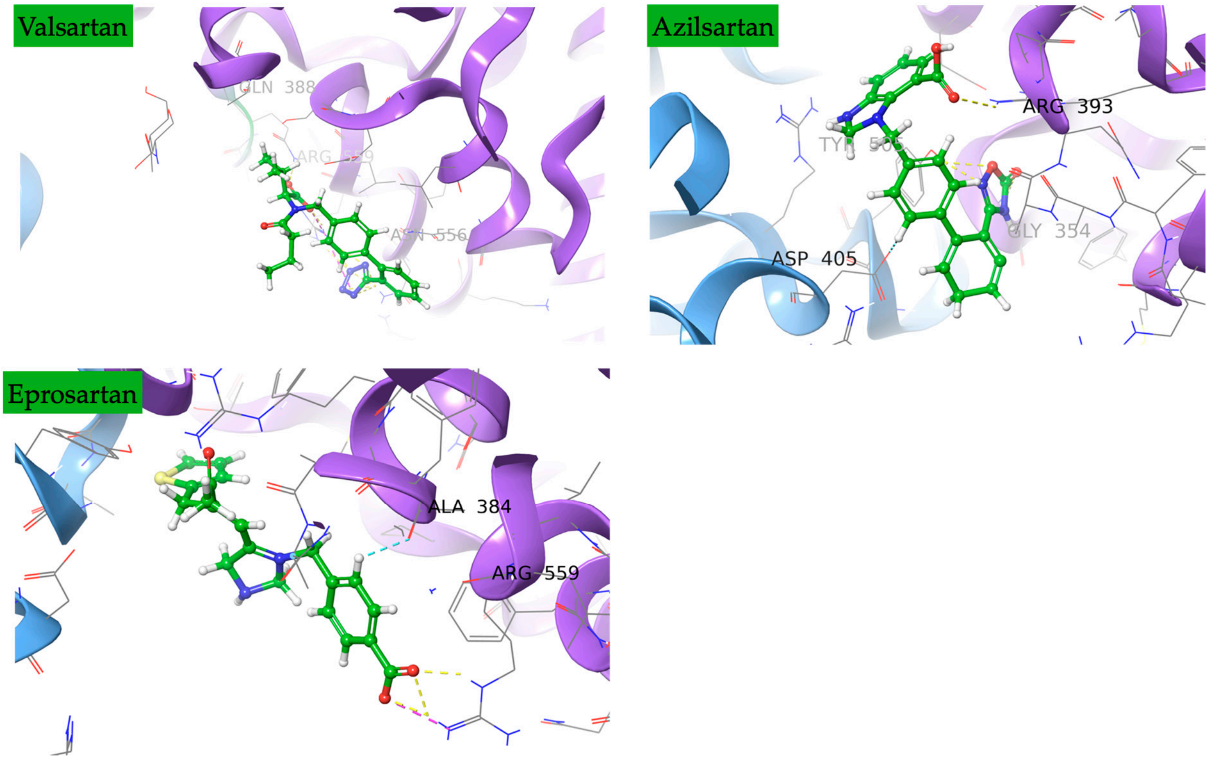

Figure S4. The IFD-derived docked poses of the complexes involving the S protein and natural products, as well as the S protein and antihypertensives, are presented for the third binding site. In the visualization, the ACE2 (chain A) is depicted with a purple ribbon, and the S protein (chain E) is shown in light blue. The third binding site is located at their interface.

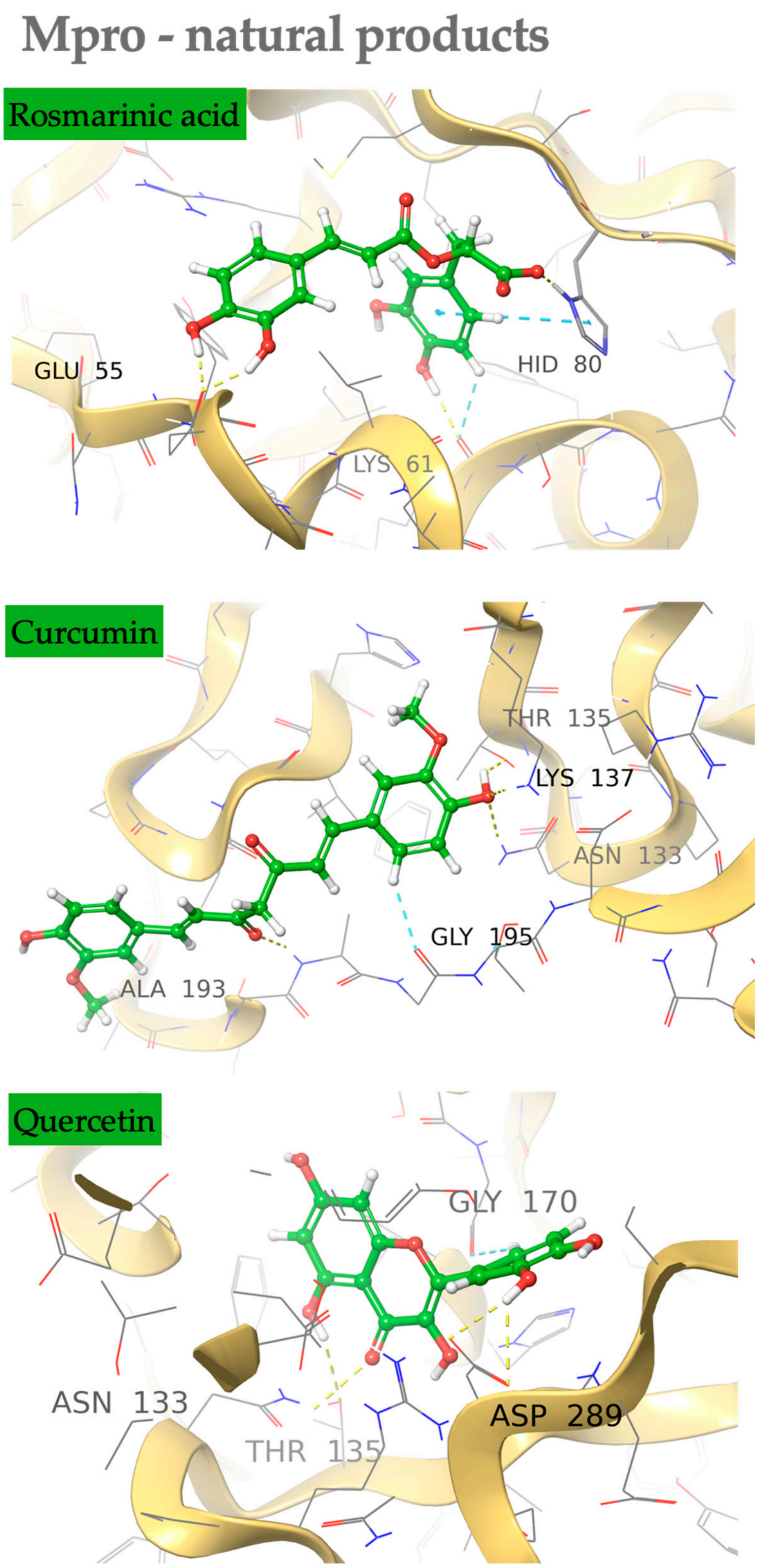

Figure S5. The predominant representative conformation of the Mpro – natural products complexes obtained through clustering analysis. The centroid represents 22%, 17% and 24% of the total simulation time for rosmarinic acid, curcumin and quercetin complexes, respectively.

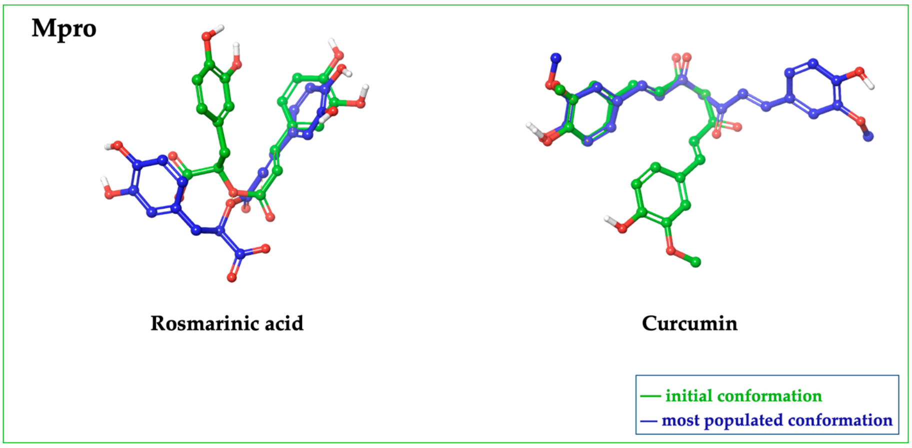

Figure S6. Superposition of the initial rosmarinic acid and curcumin conformation (green) derived from IFD to the Mpro with the centroid deduced from clustering analysis of the MD simulations (blue).

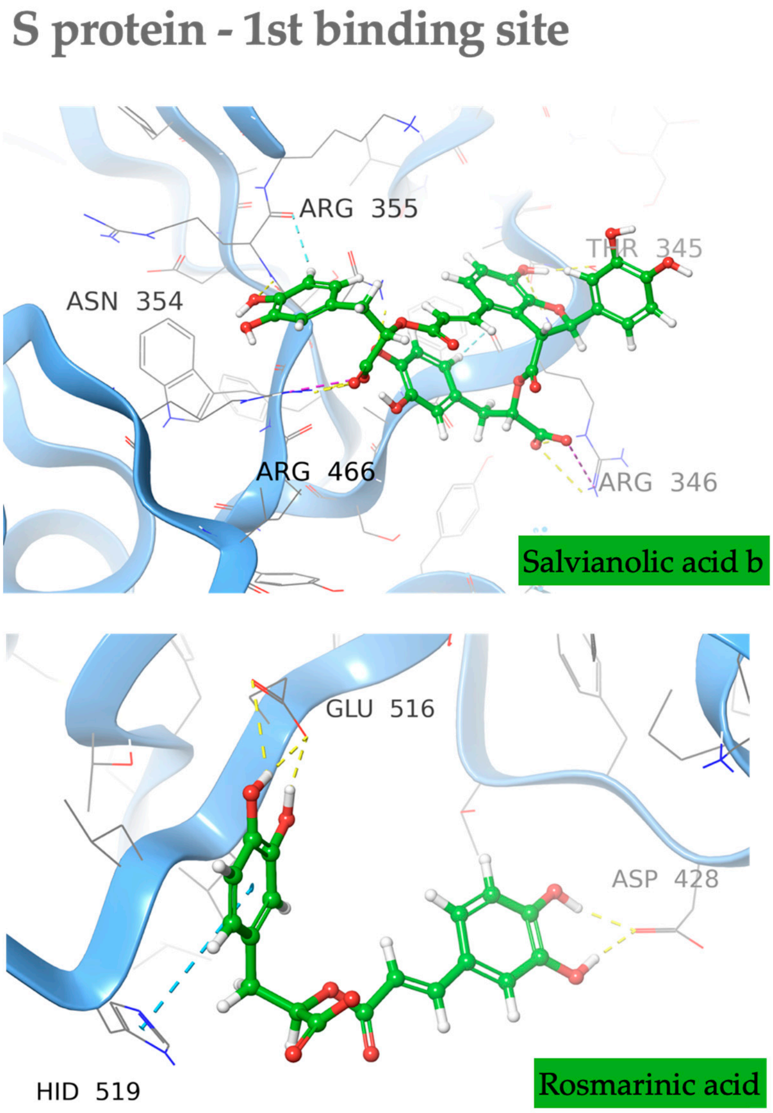

Figure S7. The predominant representative conformation of the S protein – natural products complexes obtained for the first binding site through clustering analysis. The centroid represents 22% and 19% of the total simulation time for salvianolic acid b and rosmarinic acid complexes, respectively

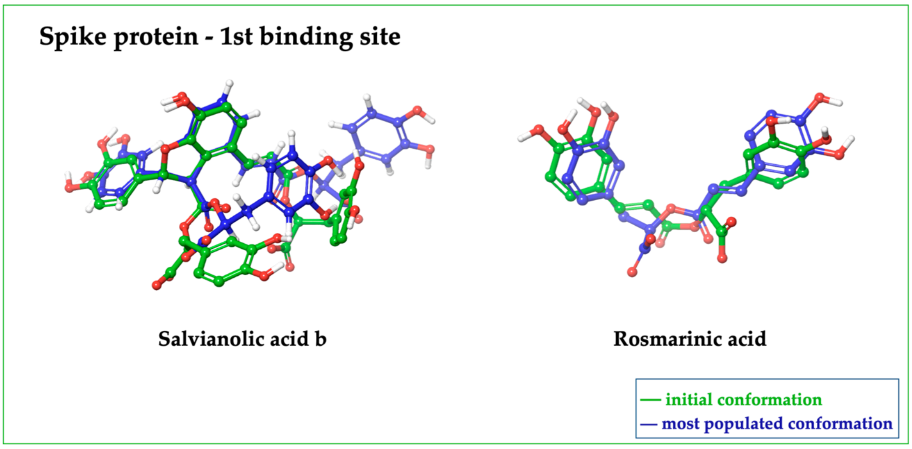

Figure S8. Superposition of the initial ligand conformation (green) derived from IFD for the first binding site of the S protein with the centroid deduced from clustering analysis of the MD simulations (blue).

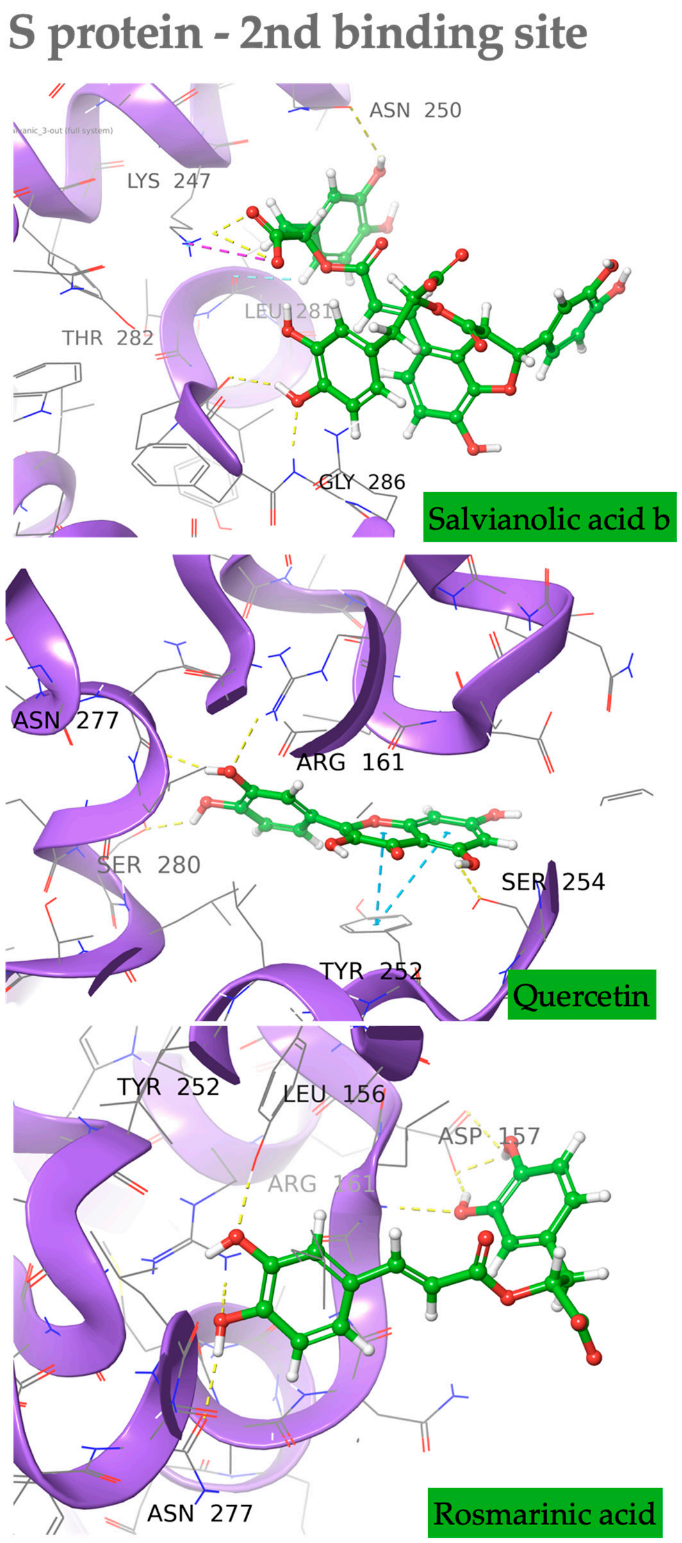

Figure S9. The predominant representative conformation of the S protein – natural products complexes obtained for the second binding site through clustering analysis. The centroid represents 26%, 40%, and 29% of the total simulation time for salvianolic acid b, quercetin and rosmarinic acid complexes, respectively.

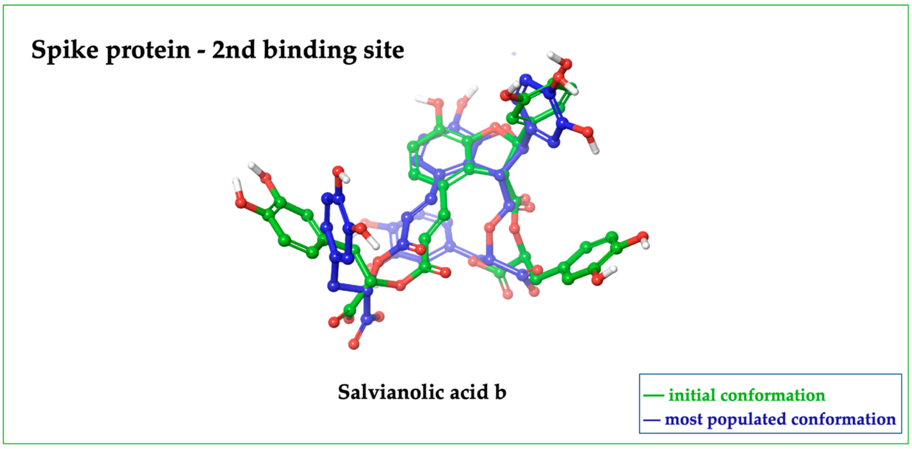

Figure S10. Superposition of the initial salvianolic acid conformation (green) derived from IFD for the second binding site of S protein with the centroid deduced from clustering analysis of the MD simulation (blue).

S protein - 3rd binding site

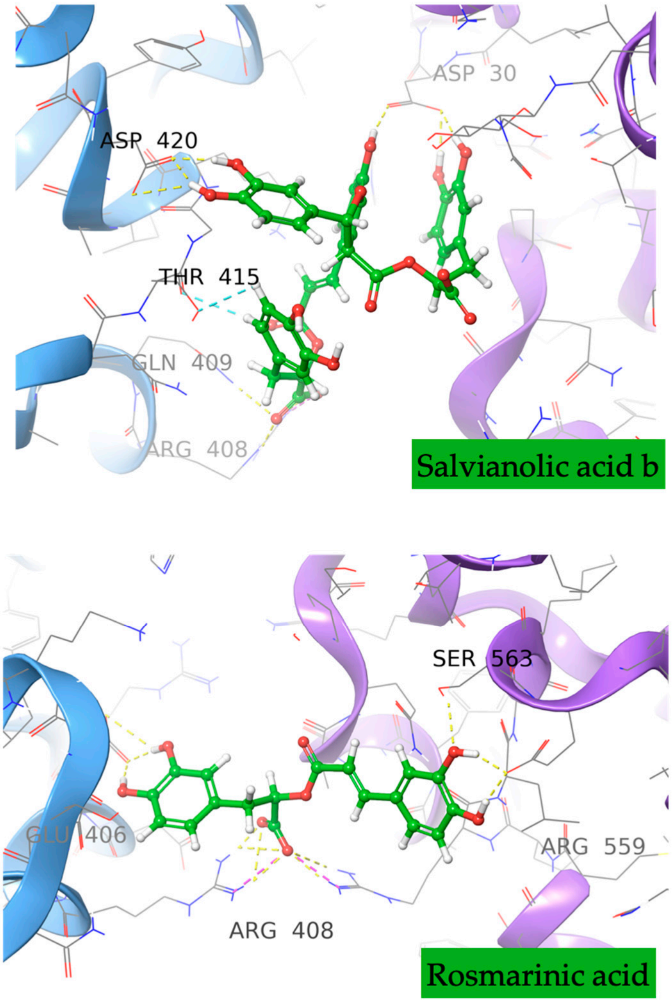

Figure S11. The predominant representative conformation of the S protein – natural products complexes obtained for the third binding through clustering analysis. The centroid represents 24% and 21% of the total simulation time for salvianolic acid b and rosmarinic acid complexes, respectively.

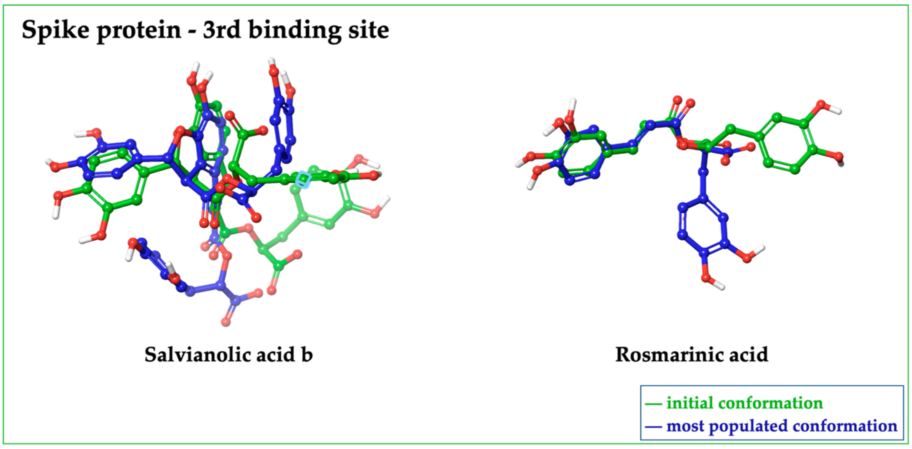

Figure S12. Superposition of the initial salvianolic acid b and rosmarinic acid conformation (green) derived from IFD for the 3rd binding site of S protein with the centroid conformation deduced from clustering analysis of the MD simulations (blue).

Table S3. The results of Induced Fit Docking (IFD) for the 116 compounds generated through a similarity search towards the binding site of Mpro (PDB ID: 6LU7) and the three separate binding sites of the S protein (PDB ID: 6M0J). The binding affinities are in units of kcal mol^{-1}.

Structure (CID)	Binding Affinity			
	Mpro	Spike protein	ACE2	New binding site
**2889**	-10.4	-6.9	-5.7	-5.7
**5099**	-13.3	-11.5	-	-7.4
**65035**	-12.0	-9.5	-7.7	-6.6
**125990**	-15.1	-12.8	-9.0	-10.8
**146723**	-11.3	-7.1	-6.7	5.4
**390474**	-11.3	-8.1	-10.0	-6.8
**439589**	-13.3	-14.7	-8.1	-7.4
**502233**	-15.4	-12.4	-9.0	-9.8
**639655**	-13.3	-11.5	-4.8	-5.9
**3012090**	-12.3	12.4	-8.0	-7.2
**5280343**	-12.0	-10.9	-7.7	-5.6
**5281614**	-11.0	-10.1	-9.5	-4.6
**5281692**	-11.2	-10.3	-9.3	-6.3
**5281792**	-11.8	-13.2	-7.7	-6.6
**5281793**	-14.0	-14.4	-7.7	-10.7
**5315614**	-13.2	-13.4	-8.2	-5.6
**5315615**	-13.3	-14.7	-4.8	-5.9
**5319772**	-11.5	-13.6	-4.8	-7.4
**5469426**	-11.3	-7.7	-6.3	-5.4
**6124301**	-13.5	-10.6	-8.2	-7.5
**6479915**	-12.3	-8.9	-8.0	-5.7
**6604740**	-12.8	-7.9	-8.1	7.4
**9841799**	-12.0	-9.7	-6.4	-6.3
**9913656**	-13.9	-9.7	-9.0	-8.0
**10237698**	-13.8	-8.9	-8.7	-8.1
**10905440**	-12.5	-9.1	-6.7	-7.1
**10958864**	-10.3	-8.5	-5.9	-6.7
**10970786**	-11.5	-9.6	-6.1	-7.4
**11495229**	-8.7	-2.1	-	-4.3
**11640287**	-11.4	-10.0	-6.7	-6.1
**11953776**	-13.7	-7.7	-8.3	-6.8

Table S3 continued

**11956645**	-12.0	-9.6	-7.3	-7.0
**11956930**	-12.7	-10.1	-7.9	-7.0
**12305312**	-11.4	-6.3	-6.2	-3.8
**12309893**	-10.6	-7.9	-5.3	-4.6
**16082534**	-11.4	-10.0	-	-6.1
**21600688**	-11.0	-6.3	-7.2	-7.0
**24862412**	-14.6	-14.3	-8.3	-8.0
**24862413**	-14.0	-13.2	-7.0	-9.4
**24884282**	-10.6	-9.1	-6.1	-5.2
**25027450**	-15.1	-13.1	-9.0	-7.5
**25201609**	-9.6	-10.9	-6.7	-8.2
**25244691**	-9.6	-10.9	-6.7	-8.2
**25245604**	-12.4	-13.0	-8.7	-7.7
**29927682**	-15.4	-9.7	-8.2	-7.5
**44437692**	-12.9	-12.3	-8.5	-6.0
**44437696**	-13.0	-11.0	-8.9	-6.7
**45934363**	-12.8	-7.9	-	-7.4
**46782412**	-11.4	-7.9	-7.9	-5.8
**46906036**	-11.1	-6.1	-	-5.5
**49849859**	-11.2	-10.6	-8.6	-5.3
**49849860**	-11.7	-10.1	-8.1	-4.7
**51352598**	-13.7	-12.3	-7.9	-10.9
**53251006**	-9.6	-7.5	-6.6	6.8
**54277438**	-12.7	-10.1	-8.2	-7.3
**54758660**	-10.7	-7.6	-9.3	-5.1
**57328179**	-12.3	-8.9	-8.0	-5.6
**59112128**	-11.0	-5.5	-5.5	-6.4
**59574960**	-9.3	-6.2	-8.4	-5.7
**69680617**	-13.8	-8.9	-8.1	-8.1
**71699430**	-8.3	-9.3	-7.0	-6.1
**71699506**	-9.1	-7.9	-6.2	-7.0
**71699507**	-10.2	-8.4	-9.2	-6.3
**73038698**	-12.6	-10.0	-7.6	-6.1
**73153047**	-12.0	-9.6	-7.7	-6.9
**74336856**	-13.7	-10.5	-9.6	-10.9
**74413535**	-11.4	-8.5	-6.9	-6.7
**74539584**	-15.1	-7.0	-10.9	-10.0
**76511959**	-11.5	-9.6	-8.4	-7.4
**88462991**	-10.7	-6.9	-7.2	-6.1
**91069955**	-11.2	-6.1	-7.2	-5.9
**91412498**	-11.5	-6.1	-6.3	-4.4
**101792073**	-11.5	-6.3	-6.1	5.1
**101792074**	-12.0	-6.3	-7.8	-5.4
**124355854**	-14.4	-9.4	-6.2	-9.0
**124487414**	-14.2	-9.5	-7.1	-9.2
**129630417**	-11.2	-9.0	-6.0	-4.7
**129648032**	-11.9	-8.3	-7.3	-5.3
**129713895**	-10.4	-	-4.9	-6.5
**131676018**	-16.5	-13.7	-8.2	8.9
**133561492**	-13.2	-11.6	-11.0	-8.1
**138535294**	-9.4	-11.4	-5.3	-4.2
**141240288**	-9.3	-11.5	-6.4	-5.3
**147170730**	-10.6	-6.5	-4.9	-2.8
**153409103**	-7.9	-	-5.1	-4.5
**153530089**	-11.6	-7.3	-4.0	-7.6

Table S3 continued

**154074200**	-11.9	-6.5	-	-5.0
**156767820**	-10.8	-	-5.6	-5.5
**156767821**	-11.0	-	-	-
**156767823**	-10.4	-5.9	-7.6	-6.9
**162641822**	-11.0	-6.5	-7.2	-6.8
**162859050**	-11.6	-8.4	-8.6	-6.0
**162859051**	-12.9	-8.4	-8.6	-6.0
**162859053**	-12.7	-10.1	-6.5	-7.0
**162905073**	-12.6	-7.5	-4.4	-6.7
**162905074**	-13.7	-7.7	-4.4	-6.8
**163001353**	-11.5	-9.6	-8.4	-7.4
**163001354**	-11.1	-10.2	-	-7.2
**163342012**	-10.6	-7.1	-9.3	-6.2
**100985949n1**	-11.1	-6.1	-5.1	-5.5
**100985950n1**	-12.0	-6.5	-5.9	5.8
**130345966n1**	-12.2	-14.8	-7.0	-5.9
**130345966n2**	-4.1	-2.7	-4.4	-2.1
**130367129n1**	-11.5	-	-5.6	-5.5
**131676119n1**	-12.0	-12.8	-7.4	-5.5
**131676119n2**	-12.0	-2.5	-3.3	-3.2
**142750760n1**	-13.8	-12.9	-8.6	-10.0
**159600901n1**	-10.8	-7.8	-9.5	-5.8
**16212154n1**	-12.0	-	-5.0	-3.8
**163332190n1**	-11.0	-6.3	-7.2	-7.0
**5284452n1**	-12.0	-10.8	-6.6	-5.6
**67304407n1**	-11.3	-6.4	-6.2	-5.7
**67304407n2**	-3.2	-2.3	-2.9	-1.1
**71311620n1**	-13.8	-8.5	-7.8	-10.0
**162859052**	-12.7	-13.6	-7.9	-7.3

-: not docked
